# Reversal of pancreatic desmoplasia by re-educating stellate cells with a tumour microenvironment-activated nanosystem

**DOI:** 10.1038/s41467-018-05906-x

**Published:** 2018-08-23

**Authors:** Xuexiang Han, Yiye Li, Ying Xu, Xiao Zhao, Yinlong Zhang, Xiao Yang, Yongwei Wang, Ruifang Zhao, Gregory J. Anderson, Yuliang Zhao, Guangjun Nie

**Affiliations:** 1 CAS Key Laboratory for Biomedical Effects of Nanomaterials and Nanosafety, CAS Center for Excellence in Nanoscience, National Center for Nanoscience and Technology, Beijing, 100190 P.R. China; 2 University of Chinese Academy of Sciences, Beijing, 100049 P.R. China; 3 Department of Chemistry, Tsinghua University, Beijing, 100084 P.R. China; 4 National Laboratory of Biomacromolecules, Institute of Biophysics, Chinese Academy of Sciences, Beijing, 100101 P.R. China; 5 QIMR Berghofer Medical Research Institute, Royal Brisbane Hospital, Herston, QLD 4029 Australia

## Abstract

Pancreatic ductal adenocarcinoma is characterised by a dense desmoplastic stroma composed of stromal cells and extracellular matrix (ECM). This barrier severely impairs drug delivery and penetration. Activated pancreatic stellate cells (PSCs) play a key role in establishing this unique pathological obstacle, but also offer a potential target for anti-tumour therapy. Here, we construct a tumour microenvironment-responsive nanosystem, based on PEGylated polyethylenimine-coated gold nanoparticles, and utilise it to co-deliver all-*trans* retinoic acid (ATRA, an inducer of PSC quiescence) and siRNA targeting heat shock protein 47 (HSP47, a collagen-specific molecular chaperone) to re-educate PSCs. The nanosystem simultaneously induces PSC quiescence and inhibits ECM hyperplasia, thereby promoting drug delivery to pancreatic tumours and significantly enhancing the anti-tumour efficacy of chemotherapeutics. Our combination strategy to restore homoeostatic stromal function by targeting activated PSCs represents a promising approach to improving the efficacy of chemotherapy and other therapeutic modalities in a wide range of stroma-rich tumours.

## Abstract (web summary)

Stromal-tumour interactions play an important role in pancreatic cancer progression. Here, they describe the development of a tumour microenvironment-responsive gold nanoparticle system incorporating all-*trans* retinoic acid (ATRA) and siRNA against heat shock protein 47 (HSP47), for use in pancreatic cancer treatment.

## Introduction

Pancreatic ductal adenocarcinoma (PDAC), the most common form of pancreatic cancer, is one of the most highly malignant tumours; the 5-year overall survival rate in patients with advanced PDAC is lower than 5%, even when treated with the standard, first-line chemotherapeutic drug, gemcitabine^1^. A major reason for the difficulty in treatment is the impaired delivery of chemotherapeutics into the tumour tissue as a result of the nearly impenetrable desmoplastic stroma, which has been not only shown to support tumour cell growth, invasion, and metastasis^2–4^, but also associated with resistance to chemotherapy and reduced patient survival^5^.

Pancreatic stellate cells (PSCs) are key mediators of the stromal compartment of PDAC. Upon activation, PSCs change from a quiescent state to an activated myofibroblast phenotype, which is accompanied by a loss of vitamin A-containing lipid droplets and excessive production of extracellular matrix (ECM) components, including collagen, fibronectin, proteoglycans and glycoproteins^5^. The thick ECM surrounding pancreatic cancer cells is thought to not only compress blood vessels to reduce hemoperfusion^6, 7^, but also restrict the access of drugs to tumour cells^8–11^. Strategies to eliminate the pathological barrier of PDAC, combined with standard chemotherapy, have shown some promising outcomes in clinical trials^12, 13^. However, in some cases, PDAC was changed to a biologically more aggressive tumour after depletion of the stroma^14–16^, indicating that restoration of the fibrotic stromal homoeostasis maybe a more appealing therapeutic approach^17, 18^.

All-*trans* retinoic acid (ATRA), an active metabolite of vitamin A, is proposed to maintain PSC quiescence by regulating the transcription of target genes after binding to cognate nuclear receptors^19, 20^. Previous studies have demonstrated that ATRA holds great potential to restore stromal homoeostatic function by inducing quiescence in activated PSCs^21–24^. Heat shock protein 47 (HSP47) is a collagen-specific molecular chaperone that is indispensable for the proper folding and secretion of collagen into the extracellular space. The chaperone also functions as a nodal hub in the regulation of the ECM network^25^. HSP47 has been extensively investigated in treating liver, pancreatic, peritoneal and dermal fibrosis^26–29^. In fact, a clinical trial aimed at ameliorating hepatic fibrosis (NCT02227459; Phase 1b/2; https://clinicaltrials.gov/ct2/show/NCT02227459) through the silencing of HSP47 has been completed. Due to its increased expression in PSCs in pancreatic cancer tissue, as well as its contribution to PDAC fibrosis^30, 31^, this chaperone is an appealing therapeutic target for reducing collagen accumulation and rescuing the desmoplastic stroma. To the best of our knowledge, neither an ATRA-loaded, nor an HSP47 siRNA-loaded nanosystem has been investigated to resolve tumour-associated desmoplasia. We hypothesises that combining these two active ingredients into one nanosystem could achieve more effective stromal modulation.

**Fig. 1:**
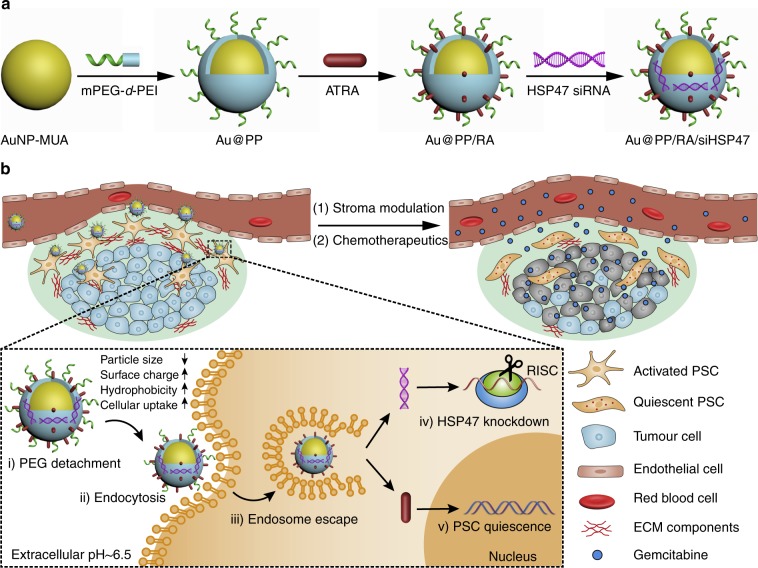
Scheme of homoeostatic restoration of pancreatic desmoplastic stroma. **a** Schematic diagram of the fabrication of the ATRA and HSP47 siRNA co-delivery system based on pH-responsive gold nanoparticles. Anionic ATRA and siRNA were electrostatic absorbed onto “sheddable” PEG-grafted polyethylenimine (PEI)-coated gold nanoparticles. **b** Schematic diagram of PSCs re-education and stroma modulation by the nanosystem. The nanosystem is “activated” (PEG shedding, size decrease, charge increase, and hydrophobic ligand exposure) in the acidic pancreatic tumour microenvironment (pHe ~6.5) and exhibits pHe and ATRA dual-enhanced cellular uptake and HSP47 knockdown in PSCs. Consequently, activated PSCs revert to quiescent phenotype and the desmoplastic stroma is homoeostatically restored, with improved blood perfusion and drug delivery

Here, we designed a pH-responsive nanosystem, based on a gold nanoparticle (AuNP), to facilitate the combinatorial re-education of PSCs by functional integration of ATRA and HSP47 siRNA (siHSP47) (Fig. 1). AuNPs are a versatile platform for the delivery of anti-cancer drugs and therapeutic oligonucleotides^32–36^ due to good biocompatibility, tunable size, scalable fashion, easy functionalization and unique optical characteristics^37, 38^. Instead of using conventional layer-by-layer assembled AuNPs for siRNA delivery^39^, we employ a facile strategy to construct PEGylated polyethylenimine (PEI)-coated AuNPs. The nanosystem exhibits a high colloidal stability under physiological conditions, thanks to the outmost PEG layer, and enhanced cellular uptake under the acidic tumour extracellular pH (pHe ~6.5)^40^ due to rapid detachment of PEG. The integrated PEI can effectively form ion-complexes with anionic ATRA and polyplexes with anionic siRNA to promote endosomal escape of the nanosystem via the “proton sponge” effect^41^. In addition to the induction of PSC quiescence, ATRA can also promote adsorptive endocytosis of the nanosystem due to the enhanced hydrophobic interaction between the hydrophobic chain of ATRA and the lipophilic cell membrane^42, 43^. Therefore, pHe and ATRA dual-enhanced cellular uptake and gene silencing are expected after PEG detachment.

We systematically investigated the combinatorial effects of ATRA delivery and HSP47 knockdown on PDAC patient-derived PSCs using our tailor-designed nanosystem. Our results show that the PSCs can be successfully reversed from the activated phenotype to a quiescent state, with abundant lipid droplets. Furthermore, the major ECM components were markedly decreased after potent silencing of HSP47 expression. Effective stromal modulation improved subsequent drug delivery and penetration in a three-dimensional (3D) PDAC stroma-rich tumour spheroid model as well as a desmoplastic PDAC xenograft tumour model. Finally, when combined with gemcitabine treatment, aggressive pancreatic tumour progression was significantly suppressed in both stroma-rich subcutaneous xenografts and orthotropic xenografts. This optimised nano-strategy, based on restoration of desmoplastic stromal homoeostasis by targeting PSCs, is a promising paradigm for the development of new combinatory regimens for pancreatic cancer treatment.

## Results

### Synthesis and characterisation of PEG-*d*-PEI

PEGylation confers nanoparticles a greater stability in the systemic circulation, but inhibits the cellular uptake and subsequent endosomal escape. This double-edged nature of PEGylation has been dubbed the “PEG dilemma”^44^. We designed PEG-detachable PEI (mPEG-*d*-PEI) in this study to circumvent the PEG dilemma by integrating a mild-acid degradable benzoic imine bond. The preparation of mPEG-*d*-PEI is facile and can be performed in two steps (Supplementary Fig. 1a). First, aldehyde group-functionalized mPEG (mPEG-CHO) was synthesised by the esterification of mPEG with *p*-formylbenzoic acid, which was verified by matrix-assisted laser desorption ionisation with time of flight (MALDI-TOF) mass spectrometry and proton nuclear magnetic resonance (^1^H-NMR) analysis (Supplementary Fig. 1b, c). Second, mPEG-*d*-PEI was obtained by the conjugation of mPEG-CHO and PEI through a Schiff base reaction in neutral, aqueous solution. The formation of the benzoic imine linker between mPEG-CHO and PEI was evident in the Fourier-transform infra-red spectrometer (FT-IR) spectrum, as denoted by an absorption peak at 1650 cm^−1^ (Supplementary Fig. 1d). The benzoic imine bond is labile and can rapidly dissociate in the slightly acidic tumour microenvironment (pHe ~6.5)^45, 46^. The low pH-triggered cleavage of the benzoic imine bond and subsequent PEG detachment were verified by ^1^H-NMR at different pH values (Supplementary Fig. 2). The aldehyde proton peak (10.01 ppm) was not observed at pH 7.4, indicating that the benzoic imine linker could not be cleaved under physiological pH. In contrast, the aldehyde proton peak of mPEG-CHO became visible after 10 min incubation at pH 6.5, suggesting a rapid PEG detachment from PEI. Moreover, most of the PEG was detached from PEI in 10 min when the pH dropped to 5.5, which corresponds to endosomal pH. These data suggest that “stealthy” PEG and PEI were successfully integrated into mPEG-*d*-PEI, which were rationally linked by a pH-sensitive benzoic imine bond.

### Preparation and characterisation of Au@PP/RA/siRNA nanosystem

**Fig. 2:**
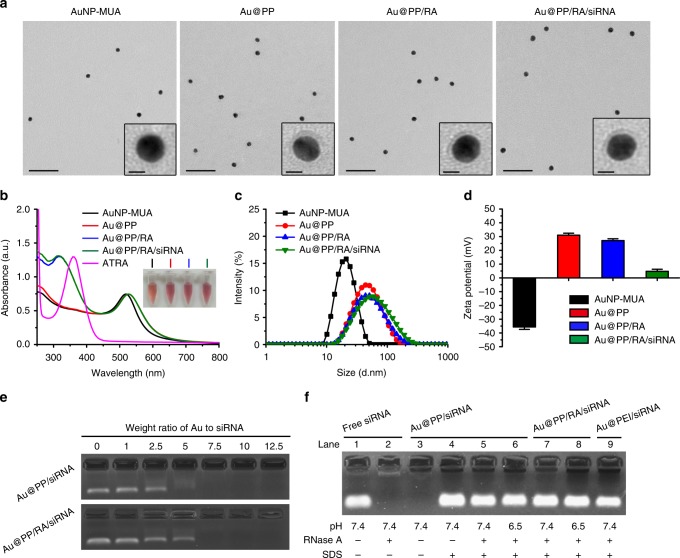
Characterisation of the Au@PP/RA/siRNA nanosystem. **a** Representative TEM images of AuNP-based nanosystems. Scale bars, 100 nm. Insets, enlarged micrographs. Scale bars, 10 nm. **b** UV–vis absorbance spectra of the indicated nanosystems and ATRA. Insets, photographs of the indicated nanosystem samples. **c** Size distribution of the nanosystems measured by dynamic light scattering (DLS). **d** Zeta potential of the nanosystems in 10 mM HEPES buffer (pH 7.4). The data are shown as the mean ± s.d. (*n* = 3). **e** Agarose gel electrophoresis retardation assay of siRNA at various w/w ratios of Au to siRNA. Complete retardation of siRNA was achieved at the w/w ratio of 7.5 for both nanosystems. **f** siRNA protection assay of different nanosystems treated with RNase A for 2 h at pH 7.4 or 6.5. Compared to the untreated lane 4 (Au@PP/siRNA without RNase A treatment, set as 100%), the remaining siRNA amounts were 81%, 77%, 84%, 77 and 60% for lanes 5–9, respectively, after RNase A treatment

The fabrication of the Au@PP/RA/siRNA nanosystem for PSC re-education is illustrated in Fig. 1. The 11-mercaptoundecanoic acid-capped AuNPs (AuNP-MUA) were prepared according to Elbakry’s protocol^39^. Transmission electron microscopy (TEM) images of AuNP-MUA revealed a typical spherical morphology with a diameter of 15 nm (Fig. 2a). The particles also exhibited a characteristic surface plasmon resonance peak at 520 nm, with a hydrodynamic diameter of 19.3 ± 1.2 nm and a negative surface charge of -35.7 ± 1.7 mV (Fig. 2b–d). AuNPs were coated with mPEG-*d*-PEI, through electrostatic interactions between the positively charged mPEG-*d*-PEI and negatively charged AuNP-MUA, to form Au@PP. The deposition of the mPEG-*d*-PEI was substantiated by TEM imaging, which shows the AuNP core surrounded by a polymer shell with a thickness of approximately 3 nm (Fig. 2a). The characteristic absorption peak was redshifted to 525 nm; the hydrodynamic diameter was increased to 41.5 ± 2.7 nm and the surface charge was reversed to 31.5 ± 1.5 mV. The prepared Au@PP exhibited good colloidal stability under physiological pH, for the particle size and polydispersity index (PDI) showed negligible changes at pH 7.4 for 24 h (Supplementary Fig. 3).

Next, we monitored the pH-responsive behaviour of Au@PP by measuring particle size and surface charge at different pH values. When the pH was lowered to 6.5, the particle size decreased to 38.0 ± 0.5 nm while the zeta potential increased to 33.7 ± 0.7 mV (Supplementary Fig. 4). A further size decrease (35.1 ± 0.2 nm) and charge increase (36.5 ± 1.2 mV) of Au@PP were observed at pH 5.5. In accordance with the ^1^H-NMR spectra of mPEG-*d*-PEI (Supplementary Fig. 2b), these variations are attributed to the rapid dissociation of the benzoic imine bond and the detachment of PEG from the Au@PP under acidic conditions. Together, these results verify our successful fabrication of a pH-responsive Au@PP nanosystem with a “sheddable” PEG layer.

ATRA was loaded into Au@PP through electrostatic interactions between the carboxyl group of ATRA and the amine groups of PEI^47^. The drug encapsulation efficiencies at different feed weight ratios of ATRA/Au were measured (Supplementary Table 1) and a weight ratio of 0.5 (approximately 18,800 ATRA molecules per AuNP) was chosen for subsequent experiments for its relatively small size (47.7 ± 3.8 nm), preferable positive charge (28.4 ± 1.3 mV), good dispersity (PDI = 0.25) and high encapsulation efficiency (65.9 ± 4.2%). After ATRA loading, a new peak at 315 nm appeared in the UV–vis spectrum of Au@PP/RA (Fig. 2b). While ATRA showed a characteristic absorption peak at 360 nm, the blue shift implied a strong electrostatic interaction between ATRA and PEI^47^. Au@PP/RA showed a pH-dependent ATRA release profile (Supplementary Fig. 5). The protonation of anionic ATRA (p*K*a ~6.0–6.5) within the ATRA/PEI ion-complexes led to the dissociation of the ionic bonds at pH values lower than the pKa^48^, which may trigger a faster release.

We next tested the siRNA condensation capability of Au@PP and Au@PP/RA using gel electrophoresis retardation assays at various weight ratios of Au to siRNA (Fig. 2e). Complete retardation of the siRNA for both Au@PP and Au@PP/RA was found at a ratio of 7.5 (approximately 230 siRNA molecules per AuNP). This ratio was used for subsequent experiments. After the siRNA loading, the UV–vis spectrum of Au@PP/RA/siRNA showed a stronger absorption at 260 nm, in comparison with Au@PP/RA (Fig. 2b). Due to highly negatively charged phosphodiester of siRNA (pKa ~1.0)^49^, less than 40% of the loaded siRNA was released from Au@PP/siRNA or Au@PP/RA/siRNA at pH 7.4, 6.5 and 5.5 after 48 h, confirming the strong electrostatic interaction between the nanosystems and siRNA (Supplementary Fig. 6). However, approximately 80% of the siRNA could be released from either nanosystem in the presence of glutathione at pH 7.4, suggesting that abundant glutathione in the cytoplasm may favour intracellular siRNA release by virtue of place-exchange reactions of thiols on gold nanoparticle surfaces^50, 51^.

The final nanosystem, Au@PP/RA/siRNA, was 50.8 ± 2.4 nm in diameter with a neutral surface charge (*ζ* = 4.8 ± 1.5 mV) and was well-dispersed (PDI = 0.27) in HEPES buffer (Fig. 2c, d and Supplementary Table 2). Finally, the siRNA protection by the nanosystem was tested by challenging it with RNase A. Free siRNA quickly degraded after RNase A treatment and could not be detected on the gel (Fig. 2f). However, approximately 80% of the siRNA bound to Au@PP or Au@PP/RA was protected from degradation under full (pH 7.4) or partial (pH 6.5) PEGylation; both nanosystems outperformed PEI-capped AuNPs (Au@PEI) that lacked PEG protection.

### Dual-enhanced cellular uptake and gene silencing in PSCs

**Fig. 3:**
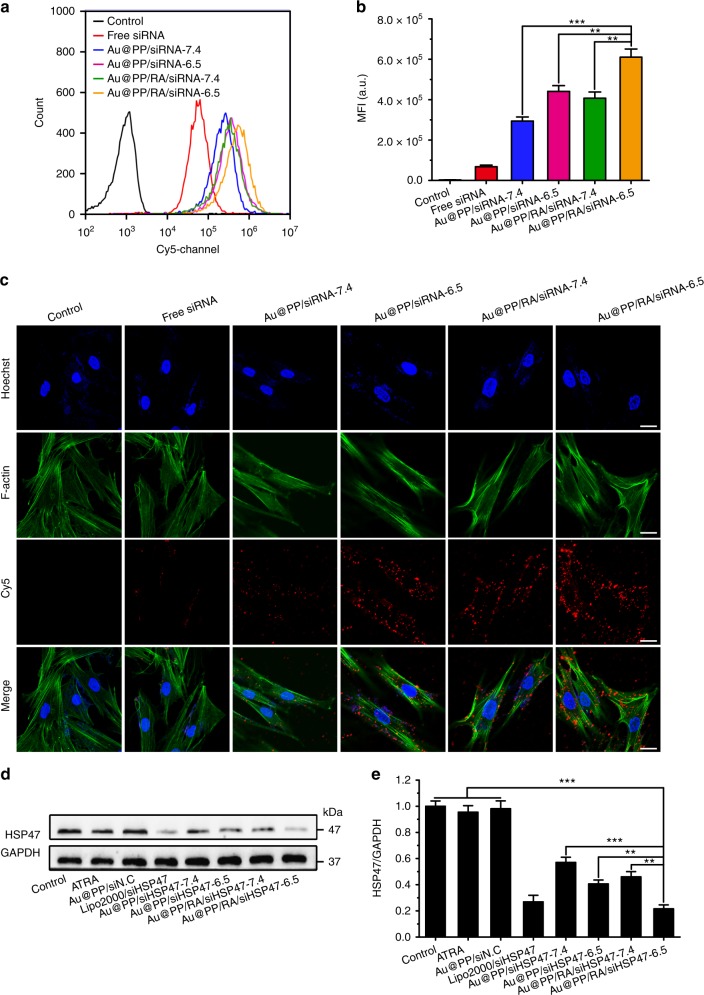
pHe and ATRA dual-enhanced cellular uptake and gene silencing. **a** Flow cytometry analysis of PSCs after incubation with Cy5-siRNA-loaded Au@PP or Au@PP/RA at pH 7.4 or 6.5 for 2 h. **b** The mean fluorescence intensity (MFI) of the different groups. The data are shown as the mean ± s.d. (*n* = 3). ^**^p *<* 0.01, ^***^p *<* 0.001 (Student’s *t* test). **c** Confocal laser scanning microscopy images of PSCs after incubation with Cy5-siRNA (red) loaded formulations at pH 7.4 or 6.5 for 2 h. F-actin was labelled with phalloidin (green) and nuclei were labelled with Hoechst 33342 (blue). Scale bars, 20 μm. **d** Western blot analysis of HSP47 protein in PSCs after treatment with the indicated formulations for 48 h at pH 7.4 or 6.5. Lipofectamine 2000 transfection agent served as a positive control (Lipo2000/siHSP47). siN.C, negative control siRNA; siHSP47, HSP47 siRNA. **e** Quantitative analysis of the normalised HSP47 protein expression (using Image J software). The data are shown as the mean ± s.d. (*n* = 3). ^**^p *<* 0.01, ^***^p *<* 0.001 (Student’s *t* test)

“Sheddable” PEG has been suggested to promote cellular uptake under local stimuli^52^. To evaluate the impact of PEG detachment on the cellular uptake of the nanosystem in vitro, PDAC patient-derived PSCs (Supplementary Fig. 7) were incubated with Cy5-siRNA-loaded Au@PP or Au@PP/RA at pH 6.5 or 7.4. The mean fluorescence intensities of the different groups were measured by flow cytometry at 2 h post-treatment. PSCs exhibited a much higher Cy5 fluorescence intensity with either Au@PP/siRNA or Au@PP/RA/siRNA treatment at pH 6.5 than at pH 7.4 (Fig. 3a, b), indicating a more efficient uptake after successful PEG detachment. Intriguingly, at the same pH, the Au@PP/RA/siRNA-treated cells displayed approximately a 1.5-fold stronger fluorescence signal than the Au@PP/siRNA-treated cells, suggesting a further promotion of the siRNA uptake by ATRA incorporation. This result implies that, after the de-shielding of PEG, the exposed ATRA facilitates adsorptive endocytosis of the nanosystem by the enhanced hydrophobic interaction between the hydrophobic chain of ATRA and the lipophilic cell membrane. Corroborating findings were obtained by examining the internalisation of Cy5-siRNA-loaded Au@PP or Au@PP/RA by confocal laser scanning microscopy (Fig. 3c), as more Au@PP/RA/siRNA entered the PSCs at pH 6.5 than Au@PP/siRNA or Au@PP/RA/siRNA at pH 7.4.

To verify pHe and ATRA dual-enhanced HSP47 gene silencing in PSCs, we screened for an effective siRNA against human HSP47 (Supplementary Table 3 and Supplementary Fig. 8), and synthesised Au@PP/siHSP47 and Au@PP/RA/siHSP47 for subsequent gene silencing experiments. Systematic comparison of the levels of HSP47 protein in PSCs after different treatments revealed that Au@PP/RA/siHSP47 treatment resulted in the highest knockdown efficiency at pH 6.5, with a 79% down-regulation of HSP47 protein compared to the untreated control (*p* < 0.001; Fig. 3d, e). Of note, ATRA treatment scarcely reduced the level of HSP47 protein, supporting that ATRA-promoted adsorptive endocytosis and subsequently enhanced gene silencing. The level of HSP47 mRNA after knockdown was examined by qRT-PCR, which further confirmed ATRA-promoted gene silencing with a remarkably down-regulated HSP47 mRNA following Au@PP/siHSP47 treatment after ATRA incorporation (Supplementary Table 4 and Supplementary Fig. 9). It is worth mentioning that, upon the internalisation of Au@PP/RA/siHSP47, the acidification of endosomes (pH ~5–6) may further strip off the residual PEG layer and maximise the membrane-disruption ability of PEI to promote endosomal escape via the “proton sponge” effect^41^, thus leading to robust gene silencing.

### Induction of PSC quiescence and inhibition of ECM production in vitro

ATRA may induce quiescence of activated PSCs, while HSP47 depletion is likely to reduce collagen deposition. We verified the combinatorial effects on PSC re-education after co-delivery of ATRA and HSP47 siRNA using Au@PP/RA/siHSP47. To mimic the acidic tumour microenvironment, these experiments were carried out at pH 6.5. The viability of PSCs was first evaluated after treatment with different concentrations of ATRA, or the various formulations. PSCs treated with ATRA and other ATRA-loaded nanosystems showed slightly less proliferation than untreated controls (Supplementary Fig. 10), which is in line with previous reports^22, 23^. Without ATRA, regardless of HSP47 depletion, no obvious cytotoxicity of Au@PP-based nanosystems was observed, indicating their favourable biocompatibility.

**Fig. 4:**
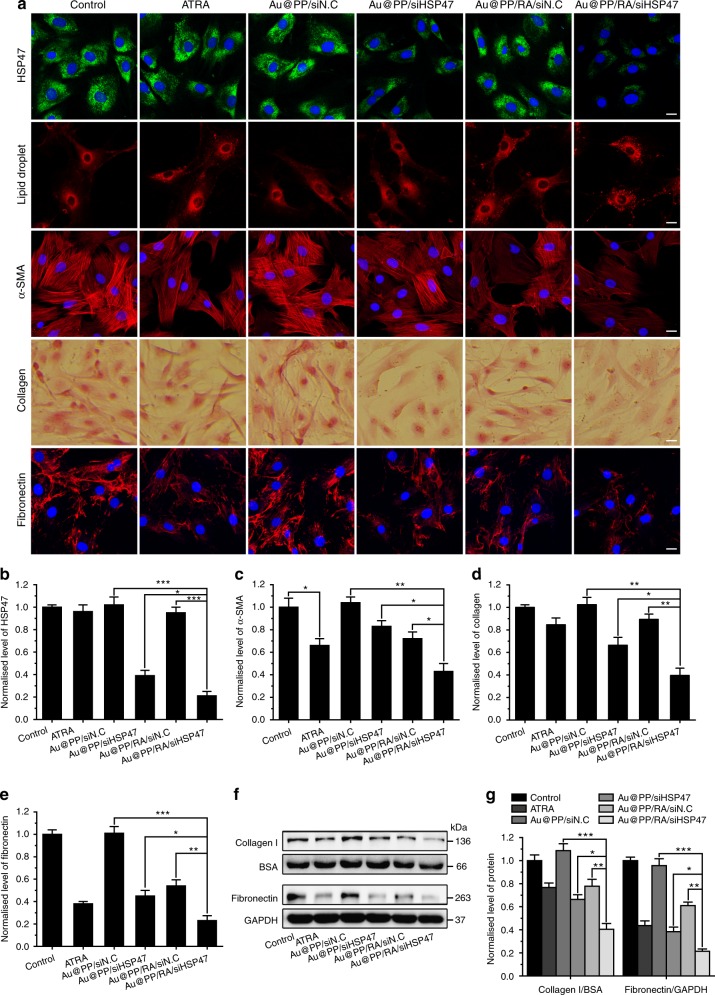
Reversal of activated PSCs and ECM reduction in vitro. **a** Immunofluorescence (IF) staining of HSP47, nile red staining of lipid droplets, IF staining of α-SMA, Sirius red staining of deposited collagen and IF staining of fibronectin in PSCs after treatment with the indicated formulations for 48 h at pH 6.5. Scale bars, 20 μm. **b** Quantification of the normalised HSP47 protein expression (using Image J software). **c** Quantification of the normalised α-SMA protein expression (using Image J software). **d** Normalised deposited collagen by measuring extracted Sirius red dye at 540 nm. **e** Quantification of the normalised fibronectin protein expression (using Image J software). **f** Western blot analysis of secreted collagen I in the PSCs culture supernatant and fibronectin in whole cell lysates after treatment with the indicated formulations for 48 h at pH 6.5. **g** Quantitative analysis of the normalised fibronectin and collagen I protein expression (using Image J software). The data are shown as the mean ± s.d. (*n* = 3). ^*^p *<* 0.05, ^**^p *<* 0.01, ^***^p *<* 0.001 (Student’s *t* test)

Next, we assessed the ability of Au@PP/RA/siHSP47 to revert activated PSCs back to the quiescent state. Immunofluorescence (IF) staining images of PSCs confirmed that treatment with Au@PP/RA/siHSP47 resulted in the most pronounced silencing of HSP47 (Fig. 4a, b), which was in accordance with the western blot results described above (Fig. 3d). Treatment with ATRA, Au@PP/siHSP47 or Au@PP/RA/siN.C (siN.C, negative control, non-targeting siRNA) significantly decreased the levels of α-SMA (marker of activated PSCs) compared to the untreated control or Au@PP/siN.C (Fig. 4a, c), suggesting a reduction in the activated phenotype. Notably, Au@PP/RA/siHSP47 treatment of PSCs resulted in the most significant reduction in α-SMA expression. Moreover, fluorescence imaging of vitamin A-storing lipid droplets (marker of quiescent PSCs) revealed that ATRA and Au@PP/RA/siN.C treatment effectively induced abundant lipid droplets in PSCs (Fig. 4a and Supplementary Fig. 11). This ability to induce PSC quiescence was further enhanced by combining HSP47 depletion, as much more lipid droplets were observed in Au@PP/RA/siHSP47-treated PSCs.

The expression levels of collagen and fibronectin, major components of the pancreatic cancer stroma, were assessed to determine the capacity of Au@PP/RA/siHSP47 to modulate the ECM. Collagen secretion from cultured PSCs was measured by Sirius red dye binding and spectrophotometry^26^. As a result of HSP47 depletion, Au@PP/siHSP47 significantly inhibited PSC collagen synthesis (Fig. 4a, d). Notably, Au@PP/RA/siHSP47 treatment elicited a greater reduction in collagen deposition through ATRA-enhanced cellular uptake of the nanosystem compared to Au@PP/siHSP47 (*p* < 0.05). Similarly, the most remarkable reduction of fibronectin occurred with Au@PP/RA/siHSP47, even though ATRA, Au@PP/siHSP47 and Au@PP/RA/siN.C treatment also greatly inhibited fibronectin production (Fig. 4a, e). Furthermore, western blot analysis of the secreted collagen I and cellular fibronectin showed a similar trend (Fig. 4f, g), further supporting the superior capability of Au@PP/RA/siHSP47 to inhibit ECM production by PSCs.

To validate whether this nano-strategy would apply to different PSC isolates, the ability of Au@PP/RA/siHSP47 to induce PSC quiescence and inhibit ECM production was further evaluated using two additional PDAC patient-derived PSCs, PSCs-2 and PSCs-3. Encouragingly, concordant with the previous results (Fig. 4a–e), Au@PP/RA/siHSP47 treatment led to a striking reduction of HSP47, α-SMA, collagen and fibronectin and induced an abundance of lipid droplets in both PSCs-2 and PSCs-3 (Supplementary Figs. 12 and 13). These data confirm that effective PSC re-education and ECM modulation can be synchronously achieved through the combinatorial nano-strategy.

### Penetration of stroma-rich 3D PDAC spheroids

The desmoplastic ECM acts as a pathological barrier which impedes drug diffusion and penetration into PDAC tissue. To test whether our nano-strategy can improve drug delivery through modulating the fibrotic stroma, we examined the penetration behaviour of small molecules in an established 3D PDAC stroma-rich spheroid (PDAC-SS) model containing pancreatic tumour cells and PSCs^53^. Panc-1, a human PDAC cell line, was chosen as the tumour cells because of its insensitivity to ATRA (Supplementary Fig. 14a). No obvious cytotoxicity from any of the formulations was observed in the Panc-1 cells (Supplementary Fig. 14b).

**Fig. 5:**
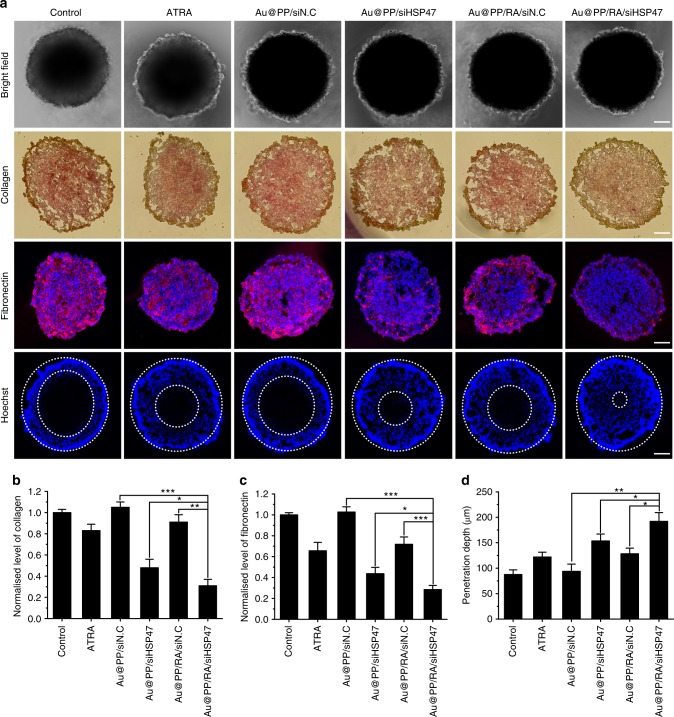
Penetration of small molecules in Panc-1/PSC stroma-rich spheroids (PDAC-SS). PDAC-SS was generated by hanging drop culture of PSCs (pre-treated with different formulations) and Panc-1 cells. **a** Representative bright field images, Sirius red staining images, fibronectin immunofluorescence images and penetrated Hoechst 33342 fluorescence images of PDAC-SS. Scale bars, 100 μm. **b**–**d** Quantification of the normalised collagen positive (Sirius red stained) area (**b**), fibronectin protein expression (**c**) and penetration depth of Hoechst 33342 (d; using Image J software). Hoechst 33342, a low-molecular weight fluorescent DNA-binding dye, was used as a probe to visualise the penetrability of PDAC-SS. The data are shown as the mean ± s.d. (*n* = 3). ^*^p < 0.05, ^**^p < 0.01, ^***^p < 0.001 (Student’s *t* test)

The generated PDAC-SS exhibited a typical spherical morphology with a radius of around 250 μm (Fig. 5a). The various treatments affected PSC synthesis of collagen and fibronectin to varying degrees in PDAC-SS; the finding of a decrease in ECM components further supports the data obtained in the 2D cell culture experiments described above (Fig. 4) with minimal staining of collagen and fibronectin in the Au@PP/RA/siHSP47-treated group (Fig. 5a–c). The penetration of small molecules into the spheroids was monitored by examining the fluorescence of Hoechst 33342, a commonly used probe in tumour penetration assays. As expected, the penetration depth of Hoechst 33342 negatively correlated with the compactness of the ECM (Fig. 5a, d). The PSCs treated with Au@PP/RA/siHSP47 resulted in the minimum ECM abundance in PDAC-SS, which exhibited the maximum Hoechst 33342 penetration depth of 192 μm, covering over 90% of the sectional area. These data suggest that the Au@PP/RA/siHSP47-based nano-strategy has considerable potential for improving drug delivery in vivo by modulating the pancreatic tumour stroma.

### Pharmacokinetics and bio-distribution in vivo

Encouraged by the effective PSC re-education and ECM inhibition by Au@PP/RA/siHSP47 in vitro, we further examined the effects of the nano-strategy in a Panc-1/PSC, co-inoculated, subcutaneous xenograft mouse model. Western blot analysis and histological staining experiments showed much higher desmoplastic activity in Panc-1/PSC than in Panc-1 tumour xenografts, as has been previously observed^54^. This is most likely due to the presence of HSP47 over-expressing PSCs (Supplementary Fig. 15), which makes this model more representative of the clinicopathological characteristics of PDAC.

**Fig. 6:**
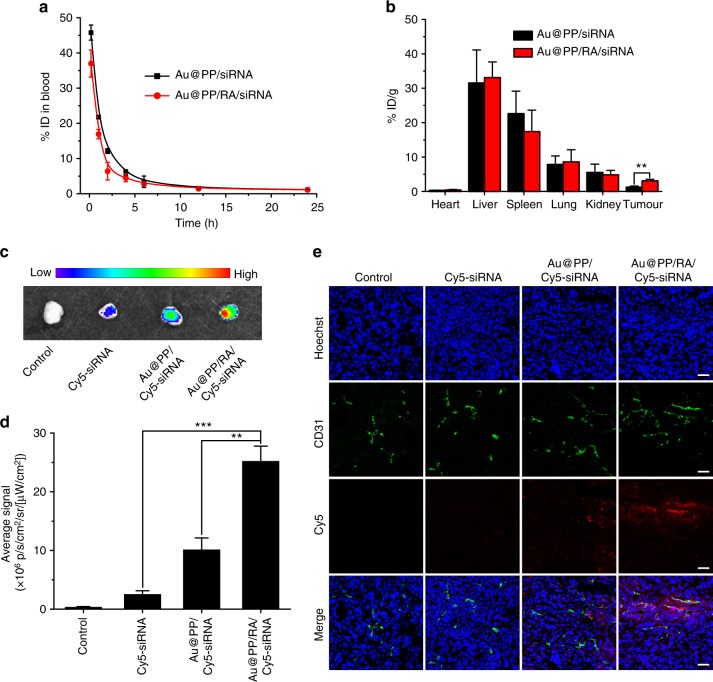
Pharmacokinetics and bio-distribution of Au@PP/RA/siRNA in vivo. **a** Pharmacokinetic curves of Au@PP/siRNA and Au@PP/RA/siRNA as analysed by the Au content in the blood using inductively coupled plasma mass spectrometry (ICP-MS), shown as the percentage of the injected dose (% ID). **b** The bio-distribution of Au@PP/siRNA and Au@PP/RA/siRNA as analysed by the Au content using ICP-MS at 24 h post-injection, expressed as the percentage of the injected dose per gram of tissue (% ID/g). **c** Ex vivo fluorescence images of representative tumours at 24 h post-injection of Cy5-siRNA, Au@PP/Cy5-siRNA and Au@PP/RA/Cy5-siRNA. **d** Quantitative analysis of the average fluorescence signal intensity in the tumours. **e** Distribution of Cy5-siRNA, Au@PP/Cy5-siRNA and Au@PP/RA/Cy5-siRNA in tumours at 24 h post-injection. Blood vessels were stained with CD31 (green) and nuclei were stained with Hoechst 33342 (blue). Scale bars, 50 μm. The data are shown as the mean ± s.d. (*n* = 3). ^**^p *<* 0.01, ^***^p *<* 0.001 (Student’s *t* test)

The pharmacokinetics and bio-distribution of Au@PP/siRNA and Au@PP/RA/siRNA were investigated in tumour-bearing mice by quantifying the gold content in the plasma, major organs and tumours using inductively coupled plasma mass spectrometry (ICP-MS). The elimination half-lives (*t*_1/2__,__elim_) of Au@PP/siRNA and Au@PP/RA/siRNA were calculated to be 9.04 and 6.21 h, respectively (Supplementary Table 5). Although Au@PP/RA/siRNA was cleared more quickly from the circulation than Au@PP/siRNA (Fig. 6a), the former exhibited greater accumulation in tumours than Au@PP/siRNA (approximately 2.5-fold, *p* < 0.01; Fig. 6b). Based on our design and the results above, this enhanced retention of Au@PP/RA/siRNA at the tumour site is presumably facilitated by ATRA, following pHe-triggered PEG detachment.

The circulation times and tumour accumulation of Cy5-siRNA, Au@PP/Cy5-siRNA and Au@PP/RA/Cy5-siRNA were also measured by quantification of Cy5 fluorescence in the blood and tumours after i.v. injection. Cy5-siRNA was cleared quickly from the circulation and showed negligible tumour accumulation at 24 h post-injection, however, the nanosystems largely extended the circulation time and improved the tumour accumulation of Cy5-siRNA (Supplementary Fig. 16 and Fig. 6c, d). Similar to the ICP-MS results, Au@PP/RA/Cy5-siRNA exhibited slightly faster blood elimination and 2.5-fold higher fluorescent signal in tumours at 24 h post-injection compared to Au@PP/Cy5-siRNA, indicating that the siRNA and nanosystem were tightly complexed in the circulation and could be synchronously delivered to tumours.

To further examine the accumulation and distribution of the nanosystems in tumours, the tumours were sectioned at 24 h post-injection of Cy5-siRNA, Au@PP/Cy5-siRNA or Au@PP/RA/Cy5-siRNA and analysed by confocal microscopy. Consistent with the ICP-MS and ex vivo imaging data (Fig. 6b, c), Au@PP/RA/Cy5-siRNA promoted more efficient Cy5-siRNA accumulation in tumours than Au@PP/Cy5-siRNA (Fig. 6e). The red fluorescence (Cy5-siRNA) separated from the green fluorescence (CD31), indicating the successful leakage of the nanosystems from the tumour vessels into the perivascular region where they could re-educate PSCs and modulate the stroma^55^. Predictably, once Au@PP/RA/Cy5-siRNA enters the acidic tumour microenvironment by the enhanced permeability and retention effect^56^, the pHe-triggered detachment of PEG exposes hydrophobic ATRA which facilitates adsorptive endocytosis of the nanosystem into adjacent PSCs and subsequently leads to effective stromal modulation.

### Stromal modulation in desmoplastic tumours and nanosafety evaluation

**Fig. 7:**
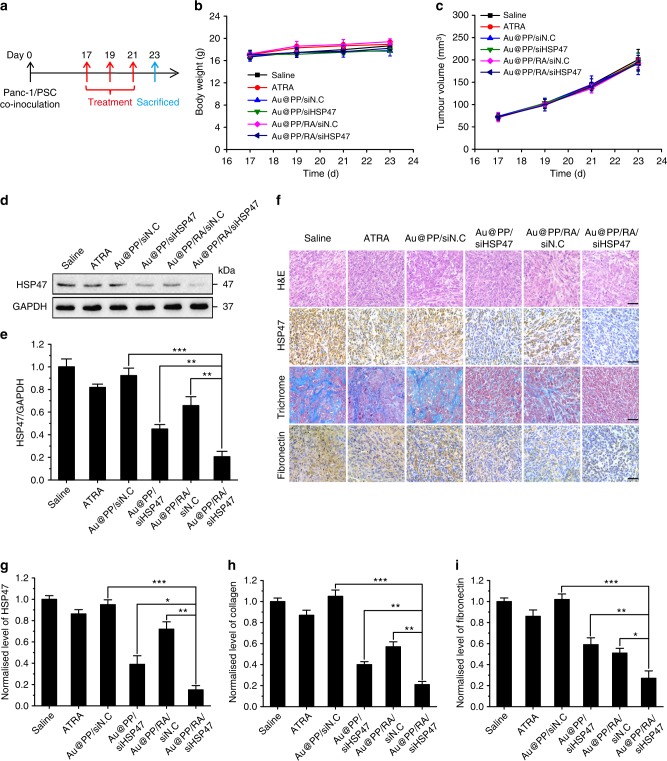
Stroma modulation in Panc-1/PSC subcutaneous xenografts. **a** Scheme of different treatment formulations for stroma modulation. The desmoplastic pancreatic subcutaneous xenografts were established by co-inoculation of Panc-1 and PSCs in BALB/c nude mice. Various formulations (ATRA: 2.4 mg/kg; siRNA: 0.97 mg/kg) were given intravenously every 2 days for three injections. **b** Body weight changes of mice during treatment. **c** Tumour growth curves during treatment. **d** Western blot analysis of HSP47 protein in tumours. **e** Quantitative analysis of the normalised HSP47 protein expression in tumours (using Image J software). **f** Histological studies with H&E, trichrome staining of collagen and immunohistochemical staining of HSP47 and fibronectin in tumour sections. Scale bars, 50 μm. **g**–**i** Quantitative analysis of the normalised HSP47 (**g**), collagen (**h**) and fibronectin (**i**) protein levels (using Image J software). The data are shown as the mean ± s.d. (*n* = 3). ^*^p < 0.05, ^**^p < 0.01, ^***^p < 0.001 (Student’s *t* test)

To further explore the stromal modulation efficacy of Au@PP/RA/siHSP47 in vivo, Panc-1/PSC tumour-bearing mice received various formulations intravenously every other day for three injections (Fig. 7a). No significant differences in body weight, tumour size and tumour weight were observed between the treatment groups (Fig. 7b, c and Supplementary Fig. 17). A steady increase in body weight in all six groups was suggestive of a good tolerance of the different formulations. However, IF staining of α-SMA revealed that Au@PP/RA/siHSP47 treatment led to a significant decrease in activated PSCs (*p* < 0.001, Supplementary Fig. 18), which benefits from its superior tumour accumulation and robust capability to re-educate PSCs. Moreover, western blot analysis of tumour tissues showed that Au@PP/RA/siHSP47 elicited an 80% reduction in HSP47 protein expression, which is considerably greater than the 55% reduction observed with Au@PP/siHSP47 (Fig. 7d, e). HSP47 protein expression was also significantly down-regulated by ATRA or Au@PP/RA/siN.C treatment, most likely due to the reduced number of activated PSCs (Supplementary Fig. 18). In addition, immunohistochemical experiments examining HSP47 corroborated the western blot results, with the greatest decrease elicited by Au@PP/RA/siHSP47 (Fig. 7f, g). Most importantly, the tumour tissues in mice receiving Au@PP/RA/siHSP47 exhibited the most significant decreases in ECM abundance (Fig. 7h, i), which was reflected by a minimal collagen and fibronectin distribution, emphasising the superior combinatorial effects of PSC quiescence and HSP47 depletion.

Previous studies have highlighted a potential systemic toxicity and immunotoxicity related to systemic siRNA delivery^57^, so we evaluated the potential side effects of Au@PP/RA/siHSP47. Serum biochemistry tests indicated no obvious liver damage or renal toxicity in any of the treatment groups at the end of the experiment (Supplementary Fig. 19). Hematoxylin and eosin (H&E) staining of the major organs revealed normal histomorphology in all groups (Supplementary Fig. 20). In addition, no significant elevation in the levels of serum IL-6 or IFN-γ (markers of inflammation) was observed in healthy BALB/c mice after the various treatments (Supplementary Fig. 21). Based on these results, we conclude that the co-delivery nanosystem can robustly restore desmoplastic stromal homoeostasis with negligible systemic toxicity and, therefore, shows great potential for combination therapy with chemotherapeutics.

### Enhanced chemotherapy after stromal modulation

The unique pathological barriers of PDAC impede drug penetration and compromise the efficacy of chemotherapy^58^. Stromal reduction is postulated to decompress tumour blood vessels, thus restoring hemoperfusion and ultimately facilitating drug delivery and penetration, which significantly enhances anti-tumour efficacy^6, 9^. With its ability to modulate desmoplastic stromal homoeostasis via the re-education of PSCs, Au@PP/RA/siHSP47 presents a promising strategy for improving the response of PDAC to chemotherapy. To test this, we first evaluated the effects of the nanosystem on tumour vessel perfusion and small molecule penetration. Au@PP/RA/siHSP47 treatment significantly increased the fraction of CD31^+^lectin^+^ vessels compared to Au@PP/siN.C (*p* < 0.05, Supplementary Fig. 22a, b), suggesting an improved vascular perfusion. Moreover, both the fluorescence intensity and penetration depth of Hoechst 33342 in the tumour tissues increased markedly after Au@PP/RA/siHSP47 treatment, even though the total vessel density did not increase significantly (Supplementary Fig. 22c, d). These data preliminarily confirm that alleviated ECM deposition by Au@PP/RA/siHSP47 can enhance tumour blood perfusion and facilitate the effective delivery and penetration of therapeutics.

**Fig. 8:**
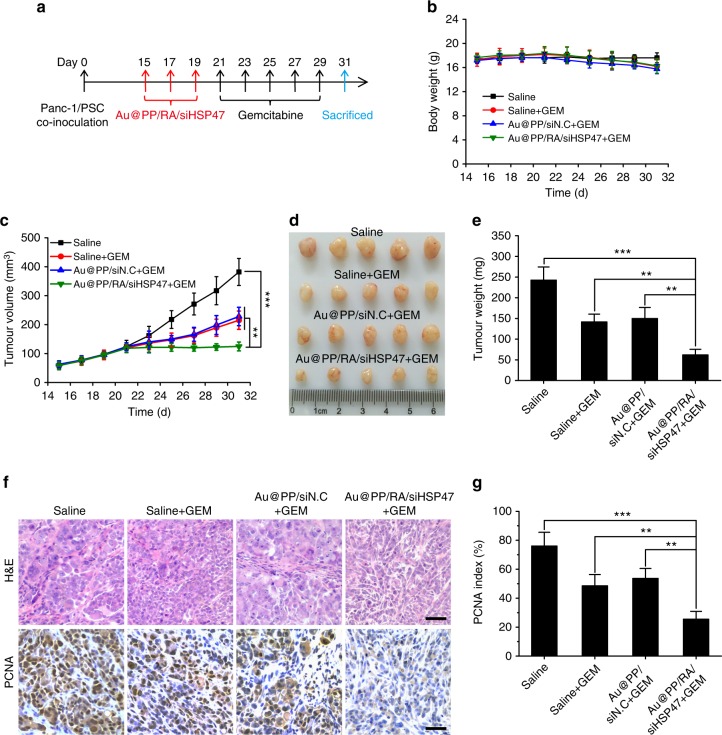
Combined treatment of Panc-1/PSC subcutaneous xenografts. **a** Scheme of combination therapy. The pancreatic tumour-bearing mice received Au@PP/RA/siHSP47 intravenously every 2 days for three injections and subsequently received gemcitabine intravenously every 2 days for five injections. **b** Body weight changes of mice during treatment. The data are shown as the mean ± s.d. (*n* = 5). **c** Tumour growth curves during treatment. The data are shown as the mean ± s.d. (*n* = 5). The mean tumour volumes were analysed using the Mann–Whitney *U* test. ^**^p < 0.01, ^***^p < 0.001. **d** Image of excised tumours. **e** Tumour weights after treatment. The data are shown as the mean ± s.d. (*n* = 5). ^**^p < 0.01, ^***^p < 0.001 (Student’s *t* test). **f** Histological studies with H&E and immunohistochemical staining of proliferating cell nuclear antigen (PCNA) in tumour sections. Scale bars, 50 μm. **g** Quantification of PCNA-positive tumour cells. The data are shown as the mean ± s.d. (*n* = 5). ^**^p < 0.01, ^***^p < 0.001 (Student’s *t* test)

We next evaluated the combinatorial effects of stromal modulation with gemcitabine (the first-line drug for PDAC) in the Panc-1/PSC subcutaneous xenograft mouse model (Fig. 8a). The combination therapy was tolerable with a slight body weight loss in all gemcitabine-treated groups (Fig. 8b), presumably due to the toxicity of chemotherapy. The tumour size increased rapidly in the saline group. In contrast, the tumour growth rate was lowered in all gemcitabine-combined groups (Fig. 8c). Encouragingly, the combination of Au@PP/RA/siHSP47 and gemcitabine resulted in the most potent anti-tumour effects with a complete halt of tumour progression, presumably due to a facilitation of drug delivery and deep tumour penetration after stromal modulation. Gemcitabine alone or Au@PP/siN.C plus gemcitabine treatment moderately reduced tumour weight, by 41.5% and 38.3%, respectively, compared to the saline-treated mice (Fig. 8d, e). What appears compelling was that treatment with Au@PP/RA/siHSP47 before gemcitabine administration significantly improved the chemotherapeutic potency, with a 74.5% reduction in tumour weight. H&E analysis of tumour sections demonstrated a less compact cell arrangement in the Au@PP/RA/siHSP47 plus gemcitabine treatment group (Fig. 8f), likely as a result of the reduction in the ECM and inhibition of tumour cell proliferation. Consistent with the tumour growth results, the treatment with Au@PP/RA/siHSP47 plus gemcitabine significantly decreased the percentage of proliferating cell nuclear antigen (PCNA)-positive cells, indicating an effective inhibition of tumour cell proliferation (Fig. 8f, g).

**Fig. 9:**
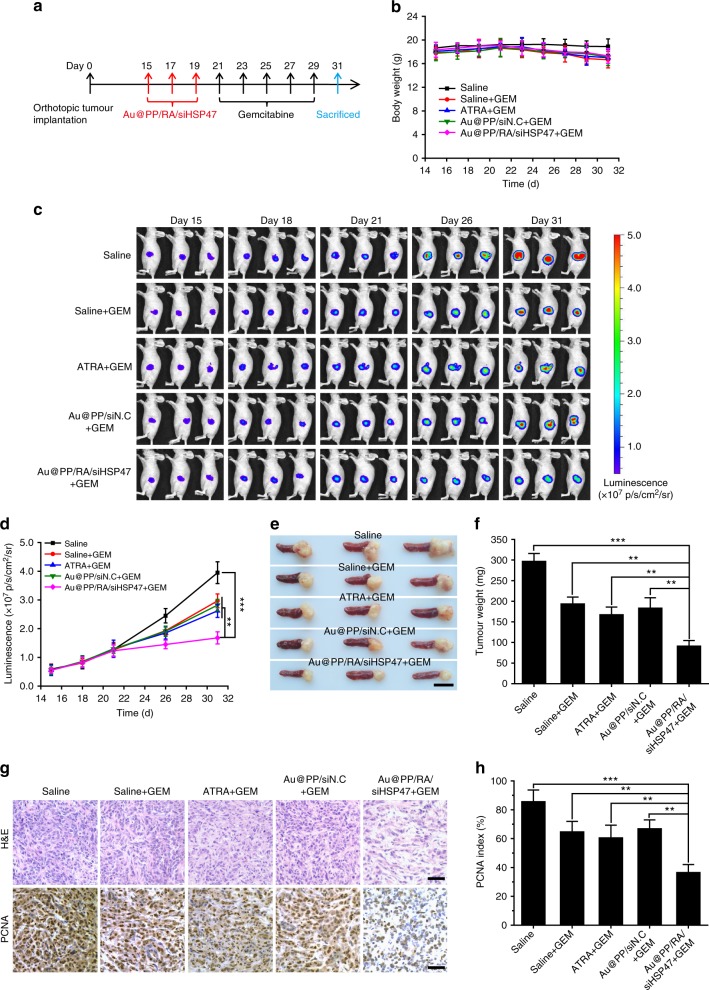
Combined treatment of Panc-1-luci/PSC orthotopic xenografts. **a** Scheme of combination therapy. The desmoplastic pancreatic orthotopic xenografts were established by co-inoculation of luciferase-expressing Panc-1 cells (Panc-1-luci) and PSCs into the pancreatic tails of BALB/c nude mice. The mice received Au@PP/RA/siHSP47 intravenously every 2 days for three injections and subsequently received gemcitabine intravenously every 2 days for five injections. **b** Body weight changes of mice during treatment. **c** In vivo whole-body bioluminescence images of mice on days 15, 18, 21, 26 and 31. **d** Tumour growth curves as determined by quantification analysis of the in vivo bioluminescence signal. **e** Image of excised tumours with spleens. Scale bar, 1 cm. **f** Tumour weights after treatment. **g** Histological studies with H&E and immunohistochemical staining of PCNA in tumour sections. Scale bars, 50 μm. **h** Quantification of PCNA-positive tumour cells. The data are shown as the mean ± s.d. (*n* = 3). ^**^p < 0.01, ^***^p < 0.001 (Student’s *t* test)

Encouraged by the superior therapeutic effect of the combination therapy in the Panc-1/PSC subcutaneous model, we further evaluated this combinatorial strategy in an orthotopic pancreatic tumour mouse model, which was established by co-inoculation of mice with luciferase-expressing Panc-1 cells (Panc-1-luci) and PSCs. This model has been rigorously demonstrated to generate a desmoplastic tumour that closely resembles human pancreatic cancer^2, 3^. Although Panc-1-luci/PSC orthotopic tumours developed an active stroma, Au@PP/RA/siHSP47 pre-treatment largely reversed this situation with a significant reduction of HSP47, α-SMA, collagen and fibronectin (Supplementary Fig. 23). In line with our previous results (Fig. 8b), a further combination therapy with gemcitabine resulted in a slight, but tolerable body weight loss in all gemcitabine-treated groups (Fig. 9a, b). In vivo bioluminescence imaging of the mice was carried out to evaluate the tumour progression throughout the experiment (Fig. 9c, d). With the rapid progression of the orthotopic pancreatic tumours, gemcitabine alone, ATRA plus gemcitabine or Au@PP/siN.C plus gemcitabine treatment only achieved moderate growth inhibition. In contrast, the Au@PP/RA/siHSP47 plus gemcitabine remarkably reduced tumour progression compared to the saline group (*p* < 0.001). Note that treatment with Au@PP/RA/siHSP47 elicited no inhibition of tumour progression within 21 days, so the enhanced chemotherapy was mainly attributed to the improved drug delivery after stromal modulation. The combination of Au@PP/RA/siHSP47 and gemcitabine resulted in the smallest tumours, with a 69.3% reduction in tumour weight (Fig. 9e, f), which is comparable to the results obtained in the Panc-1/PSC subcutaneous tumour model (Fig. 8e). Moreover, this combination therapy resulted in an obviously loose cell arrangement with the lowest percentage of PCNA-positive tumour cells (Fig. 9g, h), presumably due to the effective reduction of both ECM and tumour cells. Additionally, we assessed the tumour metastasis foci on the mesenteries. Au@PP/RA/siHSP47 plus gemcitabine treatment significantly reduced mesenteric metastases compared to gemcitabine alone, ATRA plus gemcitabine or Au@PP/siN.C plus gemcitabine treatment (*p* < 0.01; Supplementary Fig. 24), emphasising the superior anti-metastatic ability of this combination therapy. Taken together, our data strongly demonstrate that Au@PP/RA/siHSP47 possesses great potential for enhancing pancreatic cancer chemotherapy by restoring the homoeostatic stroma.

## Discussion

PDAC is a lethal cancer. An abundance of studies have highlighted the distinct pathological barriers that hinder drug delivery to pancreatic tumour cells. This phenomenon has sparked a flourish of stromal depletion strategies^59^. However, these studies have yielded contradictory preclinical and clinical responses because direct stromal depletion of PDAC runs the risk of eliminating key stromal components needed for normal tissue homoeostasis^14–16^. Thus, there remains an urgent need for more effective approaches to modulate the tumour microenvironment. Activated PSCs are the predominant collagen-producing cells with aberrant HSP47 expression and their perivascular localisation makes them attractive anti-tumour target^55^. Also, pancreatic tumours are surrounded by a more acidic microenvironment (pHe 6.2–7.0) than normal tissues because of the Warburg effect^40^, which can be utilised to design pHe-responsive nanosystems for targeting^60^.

Here, we have described a nano-enabled approach for the homoeostatic restoration of the desmoplastic stroma of pancreatic cancer by co-delivery of ATRA and HSP47 siRNA, based on pHe-responsive AuNPs. AuNPs are attractive siRNA delivery vehicles, which have been extensively exploited in in vivo studies^36, 61, 62^. However, most siRNA-AuNP nanosystems either lack shielding moieties or are decorated with permanently shielding ones. The former is neither suitable for siRNA stability, nor for adequate systemic circulation longevity, while the latter is amenable to neither cellular uptake, nor endosomal escape. We improve the siRNA-AuNP nanosystem by integrating a pHe-responsive “sheddable” PEG layer, which can circumvent the PEG dilemma by protecting the nanosystem during its blood circulation and increasing the cellular uptake in the acidic tumour microenvironment. With approximately 230 siRNA molecules per AuNP, our nanosystem possesses a relatively high siRNA-loading capacity; much higher than thiolate siRNA-attached AuNPs (30–40 siRNA molecules per AuNP)^63, 64^. Also, the anionic ATRA can easily complex with cationic PEI to form Au@PP/RA ion-complexes (18,800 ATRA molecules per AuNP), and, in turn, the hydrophobic ATRA facilitates adsorptive endocytosis of the nanosystem after PEG detachment.

Our tailor-designed Au@PP/RA/siHSP47 is primed to be “activated” in the acidic tumour microenvironment (PEG shedding, charge increase, size decrease and hydrophobic ligand exposure) and exhibits pHe and ATRA dual-enhanced cellular uptake as well as HSP47 knockdown in PSCs. Re-education of PSCs with Au@PP/RA/siHSP47 significantly decreased α-SMA expression, induced lipid droplets formation and reduced ECM production. In a desmoplastic PDAC xenograft tumour model, Au@PP/RA/siHSP47 markedly knocked down HSP47 and reduced desmoplastic activity with negligible systemic toxicity. The modulated stroma increased functional vasculature and facilitated drug delivery and penetration, thus enhancing the chemotherapeutic efficacy of gemcitabine in stroma-rich pancreatic tumours.

In summary, we have devised a nano-strategy for the homoeostatic restoration of the desmoplastic stroma of pancreatic tumours. By combining the activities of ATRA and HSP47 siRNA, our current strategy not only transforms activated PSCs into quiescent PSCs, but also effectively reduces ECM production both in vitro and in vivo. Taking advantage of the modulated stroma, the combination therapy, with tumour-directed cytotoxic treatment, leads to effective suppression of tumour progression in two desmoplastic pancreatic tumour models. Taken together, our nano-strategy to restore homoeostatic stromal function through PSC re-education holds great potential to improve the chemotherapy of pancreatic cancer and should be further explored with the goal of developing a potent PDAC combination therapy.

## Methods

### Materials

ATRA or RA, branched poly (ethylene imine) (PEI, *M*_w_ = 25 kDa), 11-mercaptoundecanoic acid (MUA), methoxy poly (ethylene glycol) (mPEG, *M*_w_ = 2 kDa), dicyclohexylcarbodiimide (DCC), and 4-(dimethylamino)pyridine (DMAP) were purchased from Sigma-Aldrich. *p*-Formylbenzoic acid, deuterated water, sodium deuteroxide (30% w/w solution in D_2_O) and deuterium chloride were obtained from Innochem (Beijing, China). Tetrachloroauric(III) acid and trisodium citrate were purchased from Sinopharm (Shanghai, China). Dulbecco’s modified eagle’s medium (DMEM) and foetal bovine serum (FBS) were acquired from Gibco BRL (Grand Island, NY, USA). Cell Counting Kit-8 (CCK-8) was purchased from Dojindo Molecular Technologies (Tokyo, Japan). siRNA and Cy5-siRNA were obtained from GenePharma Co., Ltd. (Suzhou, China). Rabbit polyclonal antibodies against GAPDH (cat# 10494-1-AP), heat shock protein 47 (HSP47, cat# 10875-1-AP), glial fibrillary acidic protein (GFAP, cat# 16825-1-AP), fibronectin (cat# 15613-1-AP), albumin (16475-1-AP), collagen type I (cat# 14695-1-AP) and cytokeratin 19 (cat# 10712-1-AP) were obtained from Proteintech (Chicago, USA). Mouse monoclonal antibodies against proliferating cell nuclear antigen (PCNA, cat# SC-56) and horseradish peroxidase (HRP)-conjugated goat anti-rabbit IgG (cat# SC-2004) were obtained from Santa Cruz Biotechnology (Dallas, USA). Rabbit polyclonal antibodies against CD31 (cat# ab28364) and α-SMA (cat# ab5694) were from Abcam (Cambridge, UK). Secondary Alexa Fluor^®^488-conjugated goat anti-rabbit IgG (H + L) and Alexa Fluor^®^ 633-conjugated goat anti-rabbit IgG (H + L) antibodies (cat# A-11034 and cat# A-21070) were purchased from Thermo Fisher Scientific (Waltham, Massachusetts, USA). Water with a resistivity above 18.2 MΩ cm was used in all experiments and all reagents were used as received without further purification.

### Synthesis of mPEG benzaldhyde (mPEG-CHO)

mPEG-CHO was prepared according to published methods^65^. Briefly, *p*-formylbenzoic acid (6 g, 40 mmol, 10 equiv), DCC (8.2 g, 40 mmol), and DMAP (1.2 g, 10 mmol) were added to a solution of mPEG (8 g, 4 mmol) in 150 mL dichloromethane (DCM). The solution was filtered after being stirred for 24 h and the filtrate was concentrated by rotary evaporation. After being dissolved in isopropanol (80 mL) and cooled at 0 °C overnight, the resulting crystals were collected by filtration and washed with isopropanol and diethyl ether to give an 80% yield.

### Preparation of AuNP-MUA

AuNPs (15 nm) were synthesised following published methods^39^. Briefly, 1% HAuCl_4_·3H_2_O (2 mL) was diluted to 200 mL and heated under reflux until boiling. 1% trisodium citrate (5 mL) was added quickly with vigorous stirring. After boiling for 10 min, the solution was filtered through a 220 nm filter to remove large aggregates. The pH of the solution was then adjusted to 11, followed by the addition of 11-MUA to a final concentration of 0.1 mg/mL. The stabilised particles were purified twice by centrifugation at 16,000×*g* for 15 min and re-suspended in 1 mM NaCl.

### Preparation of mPEG-*d*-PEI and mPEG-PEI-coated AuNPs

First, PEG-detachable PEI (mPEG-*d*-PEI) was synthesised by simply mixing PEI with mPEG-CHO at a weight ratio of 2:1 at pH 7.4 for 30 min. To prepare mPEG-*d*-PEI-coated AuNPs (Au@PP), the concentration of PEI was fixed at 1.0 mg/mL. After adding AuNP-MUA to the stirring mPEG-*d*-PEI solution, the deposition of mPEG-*d*-PEI onto the surface of the AuNP was performed for 30 min in the presence of 10 mM NaCl. The crude AuNPs were purified twice by centrifugation at 16,000×*g* for 15 min and re-suspended in 10 mM HEPES buffer (pH 7.4) with 1 mM NaCl. The concentration of gold was determined by ICP-MS.

### ATRA loading and release

ATRA was dissolved in DMSO to form a 20 mM stock solution, which was stored in the dark at −80 °C. ATRA-loaded nanoparticles (Au@PP/RA) were prepared by adding different amounts of ATRA to the above Au@PP. The formation of ion-complexes between the anionic drug ATRA and cationic PEI occurred rapidly and the solution was stirred vigorously for 30 min^47^. The nanoparticles were centrifuged twice and stored in 10 mM HEPES buffer (pH 7.4). Supernatants were collected to determine the amount of free ATRA by UV–vis spectrophotometry at 350 nm by comparison to a standard curve. The loading efficiency was calculated according to the following equation: Loading efficiency (%) = (amount of ATRA in the nanoparticles/feeding amount of ATRA) × 100.

The in vitro release of ATRA was performed in PBS at pH 5.5, 6.5 and 7.4, and monitored over time. Au@PP/RA was re-suspended in 1 mL PBS at 37 °C with gentle agitation. At the indicated time points, the suspension was centrifuged (16,000×*g*; 15 min) and the supernatant was removed and replaced with the same volume of fresh buffer. The collected supernatant was stored at 4 °C until the drug content was measured using HPLC. The mobile phase (water/acetonitrile; 15%/85%) was pumped at a flow rate of 1.0 mL/min over 10 min, with detection at 350 nm. The concentration of ATRA was determined based on the peak area at the retention time of 4.5 min, according to the calibration curve. The accumulative release of ATRA was calculated using the formula: Release percentage (%) = (amount of ATRA in PBS/amount of ATRA in the nanoparticles) × 100.

### siRNA binding, release and gel retardation assay

To determine the siRNA loading capacity, siRNA was combined with Au@PP or Au@PP/RA at various w/w ratios of Au to siRNA and incubated for 30 min to enable the formation of the complexes. After mixing with 6× loading buffer (containing SYBR Green), agarose gel electrophoresis was carried out in 1% agarose gel with TBE buffer at 120 V for 5 min. For the siRNA protection assay, siRNA was mixed with Au@PP or Au@PP/RA in HEPES buffer at pH 6.5 or 7.4 at a weight ratio of 7.5 (Au:siRNA) for 30 min. The obtained complexes were pre-treated with 0.1 mg/mL RNase A at 37 °C for 2 h, followed by incubation with 1% SDS for another 10 min before electrophoresis. The siRNA bands were visualised using a BioRad imaging system. The release of siRNA was performed in PBS at pH 7.4 (with or without 1 mM glutathione), 6.5 and 5.5 in vitro. Au@PP/Cy5-siRNA or Au@PP/RA/Cy5-siRNA was suspended in 1 mL PBS at 37 °C with gentle agitation. At varying time points, the suspension was centrifuged (16,000×*g*; 15 min), and the supernatant was removed and replaced with the same volume of fresh buffer. The collected supernatant was stored at 4 °C until the released Cy5-siRNA was determined using fluorescence spectrophotometry (*λ*_ex_ = 640 nm; *λ*_em_ = 670 nm).

### Number of molecules on each nanoparticle

The number of Au atoms per nanoparticle was 11.5 × 10^4^ for 15 nm AuNPs, according to a previous report^39^. The number of ATRA and siRNA molecules per nanoparticle was calculated based on the amount of ATRA and siRNA adsorbed onto the AuNP. It was estimated that there were approximately 18,800 ATRA and 230 siRNA molecules on each nanoparticle.

### Characterisation of nanosystem

The morphology of the nanosystems was examined by transmission electron microscopy (HT7700, HITACHI, Japan) operating at an acceleration voltage of 80 kV. Samples were prepared by depositing 6 μL of suspended nanoparticles onto a carbon-coated copper grid and air-dried before observation. The molecular weight of the products was analysed by MALDI-TOF mass spectrometry (Bruker, USA). ^1^H-NMR analysis was performed in deuterated water (D_2_O) at the required pH (adjusted by DCl or NaOD) on a Bruker Advance 400 MHz spectrometer (Bruker, USA). For FT-IR spectroscopy, freeze-dried polymer samples were pressed with KBr and scanned from 4000 to 400 cm^−1^ at a resolution of 2 cm^−1^ using a FT-IR spectrometer (Spectrum One, Perkin Elmer, USA). The size, polydispersity index and zeta potential of the samples were measured by dynamic light scattering using a Malvern Zetasizer Nano ZS90 (Malvern, UK) equipped with a He–Ne Laser at a wavelength of 633 nm and a fixed scattering angle of 173°. Three measurements each with 10 sub-runs at 25 °C were performed for each sample. UV–vis absorbance spectra of the samples were recorded using a UV–vis spectrophotometer (Lambda650, PerkinElmer, USA) in the range of 250–800 nm.

### Cell culture and animals

Human PSCs were established from pancreatic cancer surgical specimens using the outgrowth method described by Bachem et al.^66, 67^. All experiments were performed with PSCs between passages 3 and 8. The human pancreatic cancer cell line Panc-1 was obtained from the Type Culture Collection Committee of the Chinese Academy of Sciences (Shanghai, China), and Mia-PaCa-2 was obtained from the American Type Culture Collection (Manassas, VA, USA). A luciferase-expressing Panc-1 cell (Panc-1-luci) was manufactured according to the lentivirus system from Addgene. The cell lines were authenticated by short tandem repeat (STR) fingerprinting and tested negative for mycoplasma contamination. The cells were cultured in DMEM supplemented with 10% FBS, 100 U/mL penicillin and 100 μg/mL streptomycin at 37 °C in a humidified incubator of 5% CO_2_. BALB/c mice and BALB/c nude mice (female, 6–8 weeks age, 16–18 g body weight) were obtained from Vital River Laboratory Animal Technology Co., Ltd. (Beijing, China) and housed with a 12 h light–dark cycle at 22 °C and food and water ad libitum. All animal protocols were approved by the Institutional Animal Care and Use Committee of National Center for Nanoscience and Technology. The desmoplastic pancreatic xenograft tumour model was established by co-inoculation of Panc-1 and PSCs. Briefly, Panc-1 cells and PSCs (1 × 10^6^ cells each) were suspended in a 100 μL PBS and Matrigel mixture (1:1, v/v; BD, USA) and subcutaneously transplanted into the right flank of each mouse. To obtain pancreatic orthotopic xenografts, 1 × 10^6^ Panc-1-luci and 1 × 10^6^ PSCs suspended in a 50 μL PBS and Matrigel mixture (1:1, v/v) were injected into the pancreatic tails of BALB/c nude mice.

### Flow cytometry and confocal microscopy detection of cell uptake

For flow cytometry experiments, PSCs were seeded into 12-well plates at 5 × 10^4^ cells per well in 0.5 mL DMEM and allowed to grow overnight. The medium was then replaced with fresh medium (without serum) containing Au@PP/Cy5-siRNA or Au@PP/RA/Cy5-siRNA (4.2 μM ATRA, 50 nM siRNA) at pH 6.5 or 7.4, respectively. After further incubation for 2 h, the cells were collected and washed three times with PBS, then re-suspended in 200 μL PBS for flow cytometric analysis (BD Accuri C6, BD, USA). A total of 10,000 events were collected for each sample.

For the confocal microscopy, PSCs were seeded onto 8-well chambered coverglass slides (Lab-TEK, NalgeNunc) at 5 × 10^3^ cells per well, overnight, and incubated in DMEM (without serum) containing Au@PP/Cy5-siRNA or Au@PP/RA/Cy5-siRNA (4.2 μM ATRA, 50 nM siRNA) at pH 6.5 or 7.4, respectively, for 2 h. The cells were then washed three times with PBS, fixed with 4% paraformaldehyde for 15 min at room temperature, and sequentially labelled with phalloidin and Hoechst 33342, according to the manufacturer’s protocol, before imaging on a Zeiss LSM 710 confocal microscope.

### Gene silencing in vitro

PSCs were seeded in 6-well plates at a density of 5 × 10^5^ per well and cultured overnight. The medium was then replaced with fresh medium (pH 6.5 or 7.4; without serum) containing Au@PP/siHSP47 or Au@PP/RA/siHSP47 (4.2 μM ATRA, 50 nM siRNA). The cells were incubated for 6 h at 37 °C, and then the culture medium was replaced with fresh, complete DMEM for a further incubation of 42 h before the total cell protein was extracted. The protein concentration was quantified using a bicinchoninic acid protein assay kit (23225; Thermo). The cell lysates (40 μg total protein per lane) were resolved by SDS-PAGE (10% acrylamide) and then transferred to PVDF membranes. These membranes were blocked and then incubated with primary antibodies against HSP47 (1:1000) and GAPDH (1:1000) at 4 °C overnight. After being washed, the membranes were incubated with the appropriate horseradish peroxidase-conjugated secondary antibodies (1:10000) for 1 h at room temperature. The immunoreactive bands were visualised using ECL reagents. HSP47 expression was normalised to GAPDH. For uncropped scans of blots see Supplementary Figure 25.

For mRNA analysis, PSCs were treated with the different formulations at pH 6.5 for 6 h. After further incubation in fresh complete DMEM for 18 h, total RNA was extracted using the TRIzol reagent (Invitrogen, USA), and reverse transcription was carried out using the PrimeScript RT Reagent Kit (TaKaRa, Dalian, China), according to the manufacturer’s protocol. The amount of each target gene was quantified by the comparative C(T) method using β-actin as the normalisation control.

### Re-education of PSCs by ATRA and siHSP47 co-delivery in vitro

PSCs were seeded into 8-well chambered coverglass slides and treated with different formulations containing 50 nM siRNA and 4.2 μM ATRA for 6 h (pH 6.5; without serum). After supplementation with 10% FBS, the cells were further cultured for 42 h and then fixed in 4% paraformaldehyde and permeabilized in 0.2% Triton X-100 prior to blocking with 5% bovine serum albumin (BSA) for 30 min at room temperature. The primary antibodies were diluted in 1% BSA and incubated at 4 °C overnight. After being washed, the PSCs were incubated with an anti-rabbit IgG-Alexa Fluor^®^ 488 secondary antibody (1:500) or an anti-rabbit IgG-Alexa Fluor^®^633 secondary antibody (1:500) for 1 h at room temperature. The nuclei were counterstained with Hoechst 33342 and images were acquired using a Zeiss LSM 710 confocal microscope. For lipid droplets staining, PSCs with different treatments were incubated with 10 μM nile red in the dark at room temperature for 15 min. After being washed twice, lipid droplets were observed using a Zeiss LSM 710 confocal microscope. Also, lipid droplets in PSCs were examined by the fast-fading blue-green autofluorescence of retinol excited at 328 nm using an Olympus IX83 fluorescence microscope.

The deposited collagen was measured using a Sirius red dye binding assay^68^. Briefly, PSCs were seeded into 96-well plates at a density of 1 × 10^4^ per well and treated with the different formulations as described above. Collagen deposited in the wells was then stained with Sirius red dye (Solarbio S&T Co., Beijing, China), according to the manufacturer’s protocol. The bound dye was dissolved in 0.5% sodium hydroxide and the quantity of collagen was estimated by measuring the solution’s absorbance at 540 nm.

For western blot analysis of collagen I and fibronectin, PSCs were seeded in 6-well plates at a density of 5 × 10^5^ per well. After treatment with the different formulations at pH 6.5 for 6 h, the medium was supplemented with 0.5% FBS. After being cultured for a further 42 h, the cells and the corresponding culture medium in each group were collected and used for western blot analysis. BSA (1:10,000) was used as a loading control for secreted collagen I (1:1000), and GAPDH (1:1000) was used as a loading control for fibronectin (1:1000).

### Drug penetration studies with 3D PDAC stroma-rich spheroids (PDAC-SS)

3D PDAC stroma-rich spheroids (PDAC-SS) containing Panc-1 and PSCs were generated by an improved hanging drop method^53^. Briefly, after treatment with the different formulations for 6 h, PSCs were collected and mixed with Panc-1 cells at a ratio of 1:2. Hanging drop cultures were incubated for 3 days before PDAC-SS was harvested for ECM analysis and penetration studies. PDAC-SS were fixed with 4% paraformaldehyde and embedded in Tissue-Tek OCT compound (Sakura Finetek, Tokyo). Cryosections (10 μm) were prepared and collagen or fibronectin was stained as described above. For penetration analysis, PDAC-SS were exposed to 1 μg/mL Hoechst 33342 for 6 h and cryosections were visualised by confocal microscopy.

### Circulation and bio-distribution of nanosystems

Cy5-siRNA, Au@PP/Cy5-siRNA or Au@PP/RA/Cy5-siRNA (Au, 7.3 mg/kg; siRNA, 0.97 mg/kg) were injected i.v. into tumour-bearing mice (*n* = 3). Blood samples were collected at different time points post-injection. Analysis of nanosystems in the circulation was performed by measuring the gold content in the blood by ICP-MS or the fluorescence intensity of Cy5 using a Maestro in vivo imaging system (CRi Maestro^TM^, USA). Bio-distribution and accumulation analysis of nanosystems were conducted by measuring the gold content in major organs and tumours at 24 h post-injection using ICP-MS. The amount of gold was expressed as the percentage of the injected dose (% ID) or normalised to the tissue weight in grams (% ID/g). All plasma pharmacokinetic parameters were analysed by DAS software (version 2.0; Mathematical Pharmacology Professional Committee of China) using a two-compartment model.

To further determine the accumulation and localisation of Cy5-siRNA, Au@PP/Cy5-siRNA or Au@PP/RA/Cy5-siRNA in the tumours, mice were sacrificed at 24 h post-injection and the tumours were harvested for ex vivo imaging using a Xenogen IVIS in vivo imaging system. Then tumours were embedded in Tissue-Tek OCT compound, and cryosections of 10 μm thickness were prepared. Tumour vessels were identified by staining with the endothelial cell-specific marker CD31. Briefly, sections were incubated with an anti-CD31 rabbit antibody (1:50) overnight, followed by incubation with an anti-rabbit IgG-Alexa Fluor^®^ 488 secondary antibody (1:500). After labelling with Hoechst 33342 to identify nuclei, the sections were imaged using a Zeiss LSM 710 confocal microscope.

### Histology and ECM-remodelling in vivo

The ECM-remodelling efficacy of the different formulations was estimated in Panc-1/PSC co-inoculated subcutaneous xenografts. Mice were intravenously administered with saline, ATRA, Au@PP/siN.C, Au@PP/siHSP47, Au@PP/RA/siN.C or Au@PP/RA/siHSP47 (*n* = 3), when the tumour volume reached 70 mm^3^. A dose of 0.97 mg/kg siRNA (ATRA: 2.4 mg/kg) was given every other day for a total of three injections. Tumour sizes were measured every other day with a digital caliper and the tumour volume was calculated using the following formula: *V* = *L* × *W*^2^/2, where *L* is the largest tumour diameter and *W* is the smallest tumour diameter. Forty eight hours after the last treatment, the mice were euthanized. The tumour tissue and major organs were fixed overnight with 4% paraformaldehyde at 4 °C, embedded in paraffin and cut into 5 μm sections for analysis. Tissue sections were subjected to hematoxylin and eosin (H&E) staining. Tumour sections were obtained for immunohistochemical staining of HSP47 (1:50) and fibronectin (1:50). Masson’s trichrome staining for collagen was performed using standard procedures.

To determine the protein levels of HSP47 after the different treatments, excised tumours were immediately homogenised and protein extracts were used for western blot analysis.

### Haematologic examination and immune responses

Blood was collected through retro-orbital bleeding in the different treatment groups of nude mice and the plasma was recovered for blood biochemistry analysis (*n* = 3). The plasma levels of aspartate aminotransferase and alanine aminotransferase were measured to evaluate liver function. Renal function was investigated by quantifying blood urea nitrogen and creatinine levels.

To assess the immunotoxicity of siRNA after systemic delivery, healthy BALB/c mice were injected (i.v.) with a single dose of saline, siHSP47, Au@PP/RA or Au@PP/RA/siHSP47. Blood samples were collected 24 h after injection, and the serum levels of IL-6 and IFN-γ were quantified by ELISA (eBioscience, USA) according to the manufacturer’s instructions.

### Evaluation of drug perfusion and penetration in vivo

Tumour-bearing mice were administered (i.v.) Au@PP/siN.C or Au@PP/RA/siHSP47 every other day for a total of three injections (*n* = 3). At 48 h after the final injection, 0.05 mg FITC-labelled lectin (L0401; Sigma) was injected into the vein tail. Tumours were harvested at 10 min post-injection and vessel perfusion was quantified in tumour cryosections after staining with the endothelial cell-specific marker CD31. The percentage of perfused vessels was defined as the lectin^+^CD31^+^ area as a percentage of the CD31^+^ area (determined using Image J software).

The penetration of small molecules was assessed by analysing the distribution of a low-molecular weight fluorescent probe (Hoechst 33342) within the tumours (*n* = 3). At 48 h after the final injection, mice were injected with Hoechst 33342 (Sigma, 40 mg/kg) via the tail vein 1 min before euthanasia. The tumour cryosections were made and stained with the endothelial cell-specific marker CD31. Cryosections were examined by confocal microscopy and the number of CD31 positive structures was enumerated from five randomly selected fields of the stained sections.

### Combination therapy in desmoplastic xenograft tumour models

The anti-tumour efficacy of the nanosystem combined with gemcitabine was evaluated in the Panc-1/PSC co-inoculated subcutaneous xenograft tumour model. When tumour volumes reached 50 mm^3^, the mice were randomly divided into four groups (*n* = 5). Group one: i.v. administration of saline every other day for eight doses; Group two: i.v. administration of saline for three doses plus i.v. gemcitabine treatment for five doses (gemcitabine: 10 mg/kg); Group three: i.v. administration of Au@PP/siN.C for three doses plus i.v. gemcitabine treatment for five doses; Group four: i.v. administration of Au@PP/RA/siHSP47 for three doses plus i.v. gemcitabine treatment for five doses. Tumour sizes and body weights were recorded every other day during treatment. Mice were sacrificed 48 h after the last injection. Tumour tissues were fixed overnight with 4% paraformaldehyde, embedded in paraffin and cut into 5 μm sections for analysis. Tumour sections were stained with H&E. Paraffin-embedded tumour sections were subjected to immunohistochemical staining of the proliferating cell nuclear antigen (PCNA, 1:50). The PCNA index was calculated as follows: PCNA index (%) = (PCNA-positive tumour cells/all tumour cells) × 100.

In the orthotopic pancreatic tumour model, the combination treatments began two weeks after the tumour implantation (*n* = 3) and the growth of the orthotopic tumours was monitored by bioluminescent imaging of the mice at days 15, 18, 21, 26 and 31. On day 21, the ECM-remodelling efficacy of different pre-treatments (saline, ATRA, Au@PP/siN.C and Au@PP/RA/siHSP47) were analysed by western blot and histological staining experiments as decirbed above. At the end of the experiment, the mice were euthanized, and the tumour xenografts were excised and weighed. For bioluminescence imaging, d-luciferin potassium salt (150 mg/kg) was intraperitoneally injected and the mice were imaged using a Xenogen IVIS in vivo imaging system after 10 min.

### Blinding

All experimental procedures and the quantification of results, including injections, isolation of the tumours or organs and tissue histological analysis, were carried out by two independent researchers. Discordant cases were assessed by a third researcher, and a consensus was reached.

### Statistical analysis

All the results are presented as mean ± s.d. Data were analysed using Student’s *t* test or one-way analysis of variance, except where otherwise noted. Tumour volumes were compared using the Kruskal–Wallis test followed by the Mann–Whitney *U* test. Differences between groups were considered statistically significant when the two-sided *p* < 0.05. Statistics were calculated using SPSS version 19.0 software.

## Electronic supplementary material

Supplementary Information

## Data Availability

The data that support the findings of this study are available within the paper and its Supplementary Information files, or are available from the corresponding authors upon reasonable request.
