# Supplementary material for: Reversal of pancreatic desmoplasia by re-educating stellate cells with a tumour microenvironment-activated nanosystem
Source: Nat Commun. 2018 Aug 23;9:3390. doi: 10.1038/s41467-018-05906-x (PMC6107580; doi:10.1038/s41467-018-05906-x)
Supplement: Supplementary file 1 — Supplementary Information [file 41467_2018_5906_MOESM1_ESM.pdf]

Supplementary Information

Reversal of pancreatic desmoplasia by re-educating stellate cells with a tumour microenvironment-activated nanosystem

Han et al.

Supplementary Table 1. Characterization of Au@PP/RA ion-complexes

Groups	RA/Au ratio (w/w)	Loading efficiency (%, w/w)	Particle size (nm)	PDI	Zeta potential (mV)
**RA/Au-0.1**	0.1	88.2±4.5	43.4±4.6	0.22	31.4±0.9
**RA/Au-0.2**	0.2	79.7±3.8	44.0±3.8	0.23	31.2±1.5
**RA/Au-0.5**	0.5	65.9±4.2	47.7 ±3.8	0.25	28.4±1.3
**RA/Au-1**	1.0	43.1±2.8	68.1±5.8	0.36	26.7±2.7
**RA/Au-2**	2.0	38.7±1.7	87.4±5.4	0.39	25.7±3.1

Encapsulation efficiency was calculated based on UV spectrophotometry. Size and charge were collected by DLS measurements after samples were reconstituted in 10 mM HEPES buffer (pH 7.4). RA/Au-0.5 was chosen for subsequent experiments. The data are presented as the mean ±s.d. (n = 3).

Supplementary Table 2. Characterization of nanosystems prepared in this study

**Groups**	**Particle size (nm)**	**PDI**	**Zeta potential (mV)**
**AuNPs-MUA**	19.3 ± 1.2	0.11	-35.7 ± 1.7
**Au@PP**	41.5 ± 2.7	0.23	31.5 ± 1.5
**Au@PP/RA**	47.7 ± 3.8	0.25	28.4 ± 1.3
**Au@PP/RA/siRNA**	50.8 ± 2.4	0.27	4.8 ± 1.5

Samples were prepared in 10 mM HEPES buffer (pH 7.4) and data were collected by DLS measurements. The data are presented as the mean ± s.d. (n = 3).

Supplementary Table 3. Sequences of siRNA against human HSP47

Type	Sequence
**siHSP47-A sense**	5’ GCAGCAAGCAGCACUACAATT 3’
**siHSP47-A antisense**	5’ UUGUAGUGCUGCUUGCUGCTT 3’
**siHSP47-B sense**	5’ CCACACUGGGAUGAGAAAUTT 3’
**siHSP47-B antisense**	5’ AUUUCUCAUCCCAGUGUGGTT 3’
**siHSP47-C sense**	5’ GGCCUAAGGGUGACAAGAUTT 3’
**siHSP47-C antisense**	5’ AUCUUGUCACCCUUAGGCCTT 3’
**siN.C sense**	5’ UUCUCCGAACGUGUCACGUTT 3’
**siN.C antisense**	5’ ACGUGACACGUUCGGAGAATT 3’

Supplementary Table 4. List of qRT-PCR primers

Name	Primer sequence
**β-actin-F**	GCCAACACAGTGCTGTCTGG
**β-actin-R**	GCTCAGGAGGAGCAATGATCTTG
**HSP47-F**	CAGTGGAGAACATCCTGGTG
**HSP47-R**	CCGTGGAGTTGCTGAGTGA

Supplementary Table 5. Plasma pharmacokinetic parameters of the nanosystems

**Group**	$t_{1/2,\ dist}$**(h)**	$t_{1/2,\ elim}$**(h)**	***Cl*** **(L h^{-1} kg^{-1})**	$AUC^{0\text{-}\infty}$**(mg h L^{-1})**
**Au@PP/siRNA**	0.95	9.04	0.04	175.84
**Au@PP/RA/siRNA**	0.33	6.21	0.06	128.11

Mice were *i.v.* injected with Au@PP/siRNA or Au@PP/RA/siRNA (Au: 7.3 mg kg^{-1}). Gold concentrations in plasma were fitted to a two-compartment model. $t_{1/2,\ dist}$, distribution half-life; $t_{1/2,\ elim}$, elimination half-life; *Cl*, plasma clearance; $AUC^{0\text{-}\infty}$, total area under curve.

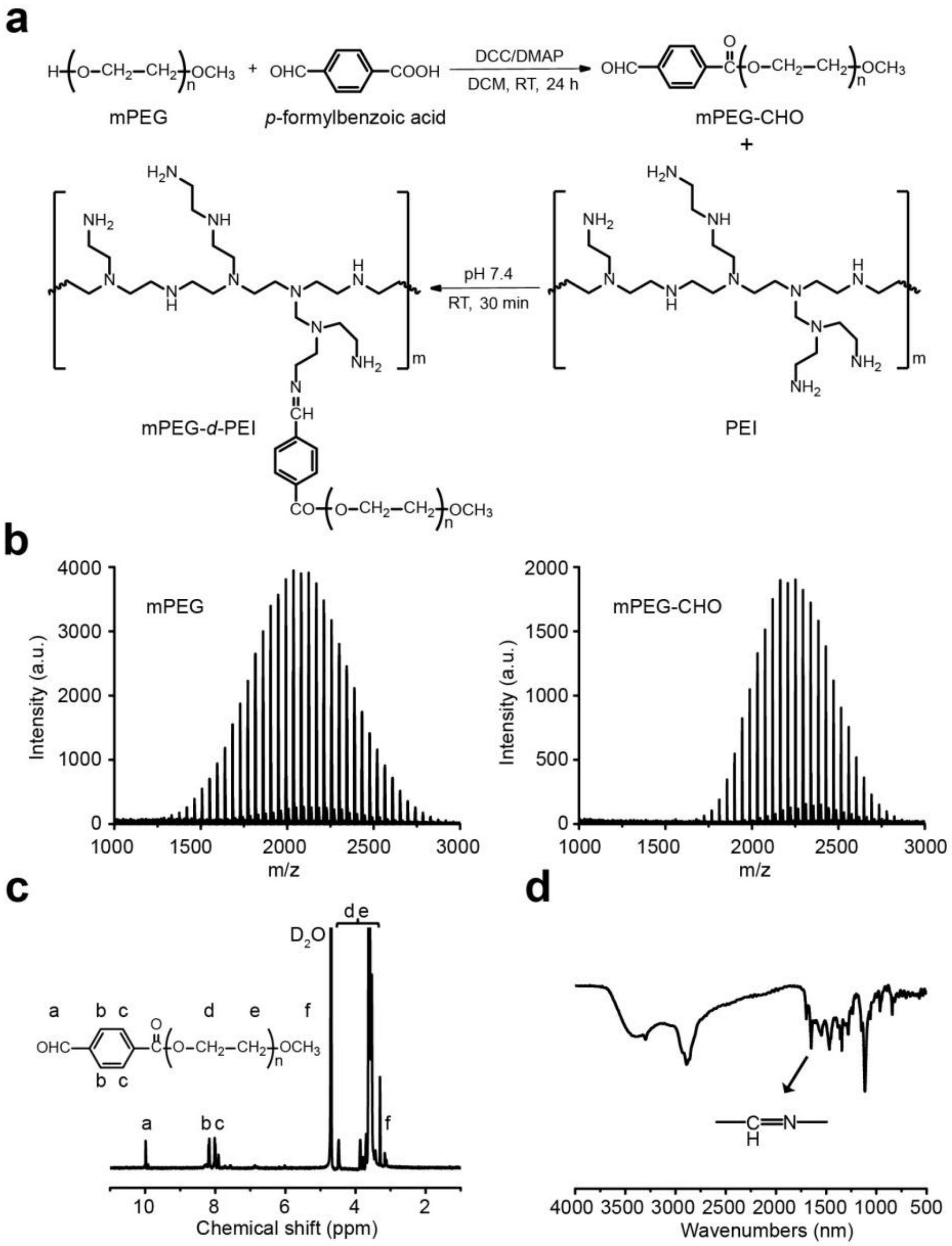

Supplementary Figure 1. Synthesis and characterization of PEG-*d*-PEI. (a) Synthetic scheme of mPEG-*d*-PEI with a pHe-labile benzoic imine bond. (b) MALDI-TOF mass spectra of mPEG and mPEG-CHO. The average molecular weights of mPEG and mPEG-CHO are 2070 and 2200, respectively. (c) ^{1}H-NMR spectrum of mPEG-CHO in deuterated water (D_2O). The ^{1}H-NMR spectrum exhibited all the characteristic proton peaks corresponding to mPEG-CHO. (d) FI-IR spectrum of mPEG-*d*-PEI. The characteristic IR band of the imine peak at 1650 cm^{-1} is clearly visible in the spectrum of mPEG-*d*-PEI.

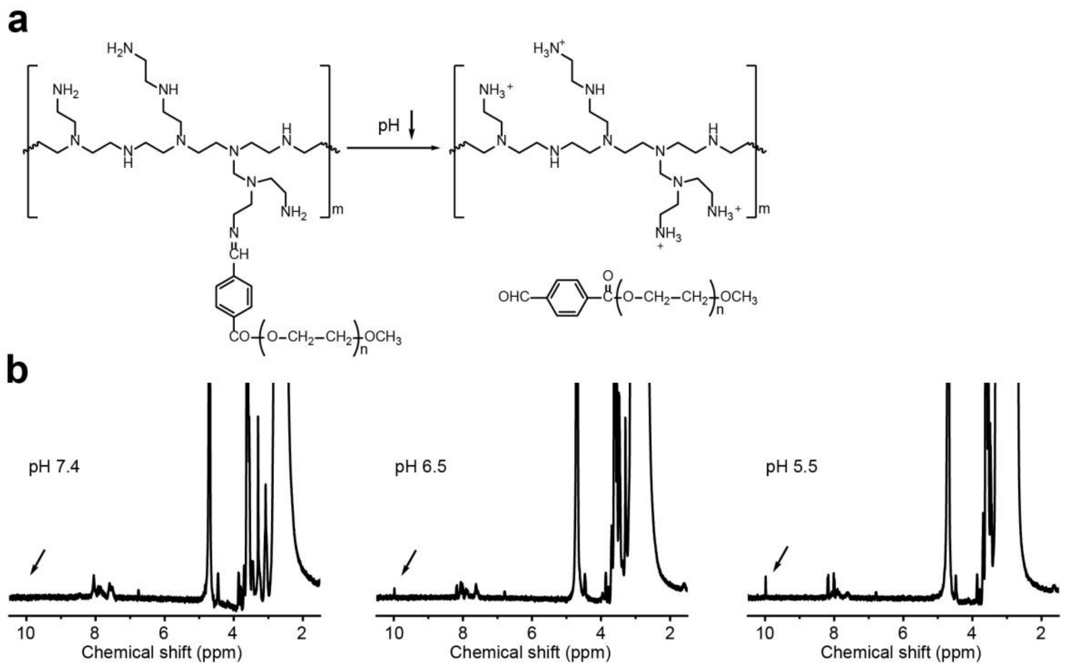

Supplementary Figure 2. pH-triggered PEG detachment from mPEG-*d*-PEI. (a) Schematic diagram of the low pH-triggered benzoic imine bond disassociation and PEG detachment from mPEG-*d*-PEI. (b) ^{1}H-NMR spectra of mPEG-*d*-PEI in (D_2O) at pH 7.4, 6.5 and 5.5. The samples were measured at 10 min post pH adjustment. The arrows indicate the aldehyde proton peaks. Based on the integral ratio of the aldehy deproton peak (10.01 ppm) to aromatic proton peak (8.21 ppm), it was estimated that 35% and 85% of PEG was disassociated at pH 6.5 and 5.5, respective-ly.

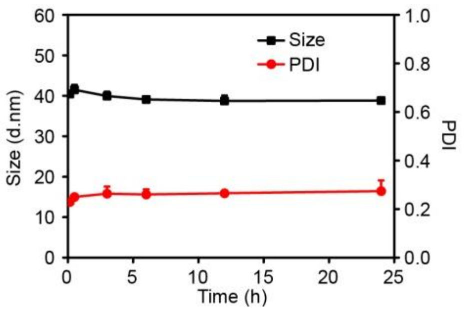

Supplementary Figure 3. Colloidal stability of Au@PP. Au@PP was dispersed in 10 mM HEPES (pH 7.4) and data were collected at different time points for over 24 h by DLS measurements. The data are presented as the mean ± s.d. (n = 3).

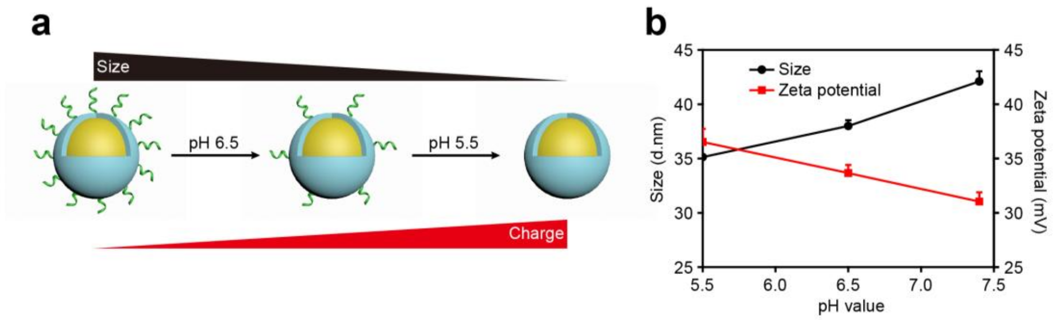

Supplementary Figure 4. pH-triggered PEG detachment from Au@PP. (a) Schematic diagram of the low pH-triggered progressive de-shielding of PEG and the subsequent changes in size and charge of Au@PP. (b) pH-responsive size and zeta potential changes of Au@PP in 10 mM HEPES at pH 7.4, 6.5 or 5.5. Data were collected at 10 min post pH adjustment by DLS measurements. The data are presented as the mean ± s.d. (n = 3).

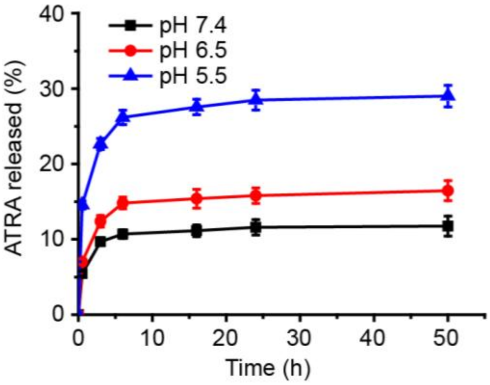

Supplementary Figure 5. ATRA release profile. Au@PP/RA was suspended in PBS buffer at pH 7.4, 6.5 or 5.5 and the released ATRA was measured by HPLC. The data are presented as the mean ± s.d. (n = 3).

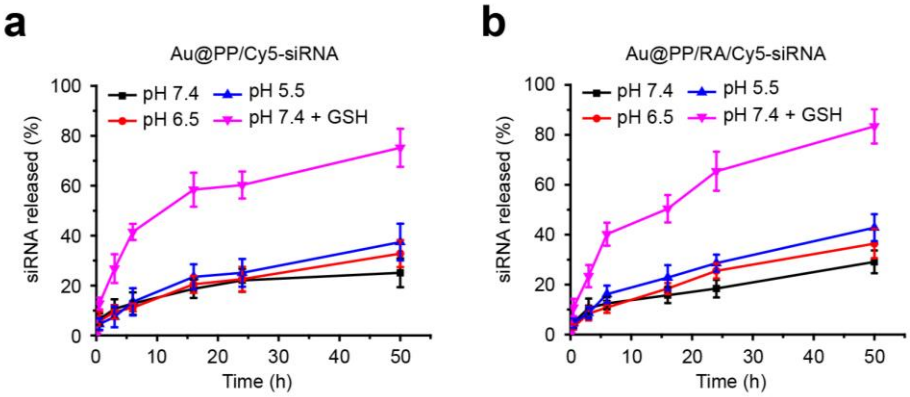

Supplementary Figure 6. The release profile of siRNA. Au@PP/Cy5-siRNA (a) or Au@PP/RA/Cy5-siRNA (b) was suspended in PBS at pH 7.4 (with or without 1 mM GSH), 6.5 or 5.5. The siRNA release was determined using fluorescence spectrophotometry. The data are presented as the mean ± s.d. (n = 3).

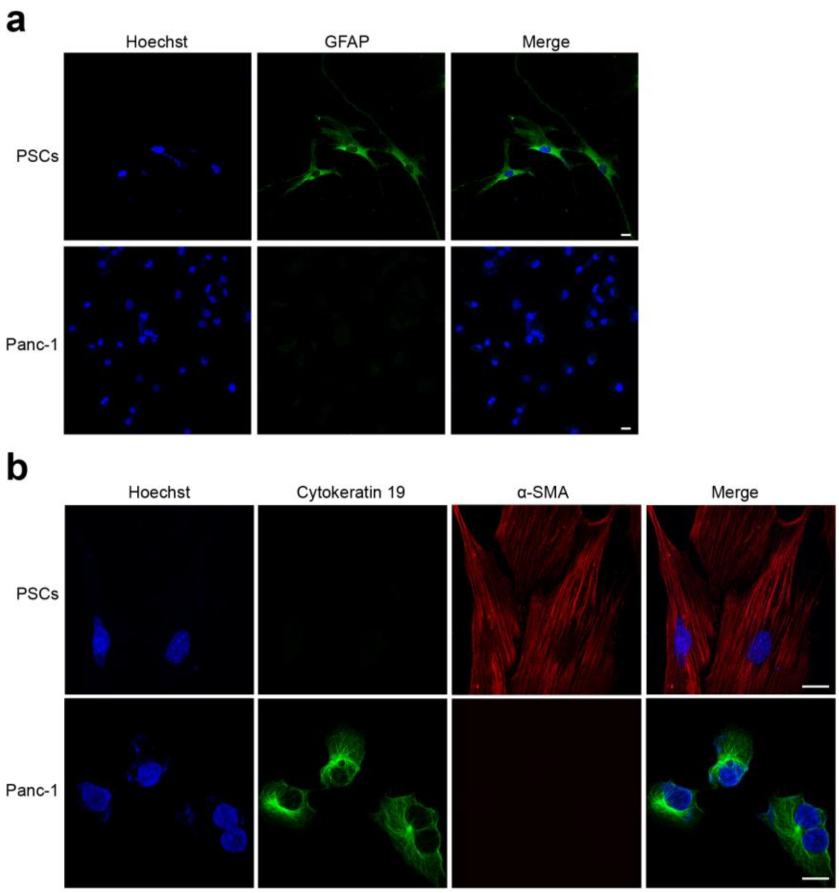

Supplementary Figure 7. Identification of human PSCs isolated from a PDAC patient specimen. The PSCs have a phenotype consistent with activated PSCs, as shown by immunofluorescent staining for the myofibrolastic markers glial fibrillary acidic protein (GFAP) (a) and α-SMA (b). Pancreatic cancer cells (Panc-1) expressed the epithelial marker cytokeratin 19, but not GFAP and α-SMA. Nuclei were labeled with Hoechst 33342. Scale bars, 20 μm.

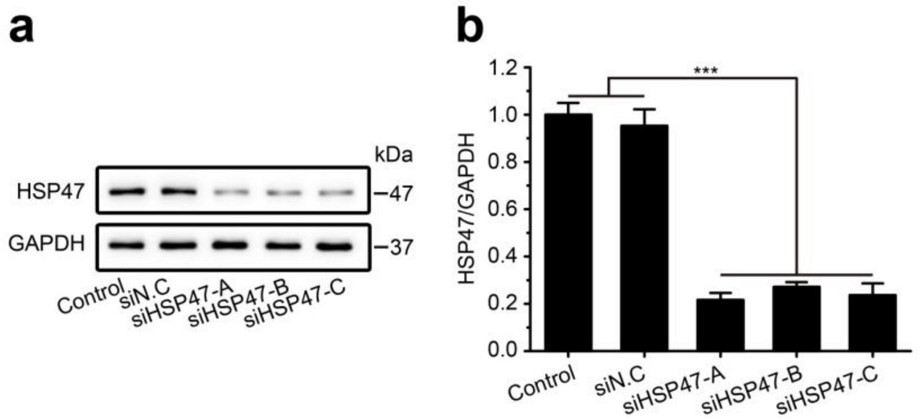

Supplementary Figure 8. Screen of siRNA sequences against human HSP47. (a) The knockdown of HSP47 mediated by different HSP47 siRNA (siHSP47) sequences. PSCs were transfected using lipofectamine-2000 (50 nM siHSP47) according to a standard protocol. Western blot analysis was conducted to compare the knockdown efficacy. (b) Quantitative analysis of the normalized HSP47 protein levels (using Image J software). siHSP47-A was chosen for subsequent experiments. The data are presented as the mean ± s.d. (n = 3). ***$p < 0.001$ (Student's *t*-test).

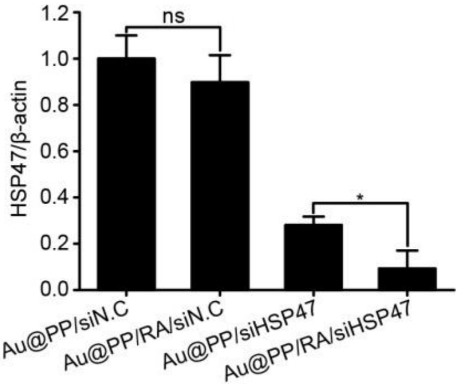

Supplementary Figure 9. HSP47 mRNA transcription levels in PSCs treated with the indicated formulations at pH 6.5. β-actin was used as an internal normalization control. The data are presented as the mean ± s.d. (n = 3). ns, no significant difference. *$p < 0.05$ (Student's *t*-test).

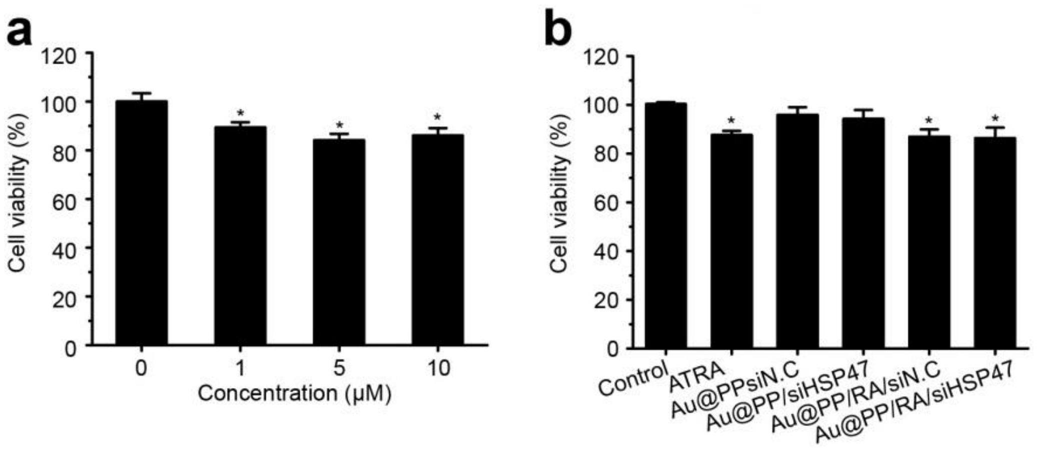

Supplementary Figure 10. Cell viability of PSCs. (a) PSCs were incubated with different concentrations of ATRA for 48 h, and the cell viability was evaluated by CCK-8 assay. (b) PSCs were treated with the indicated formulations at pH 6.5 for 48 h, and the cell viability was evaluated by CCK-8 assay. The data are presented as the mean ± s.d. (n = 3). **p* < 0.05 (Student's *t*-test).

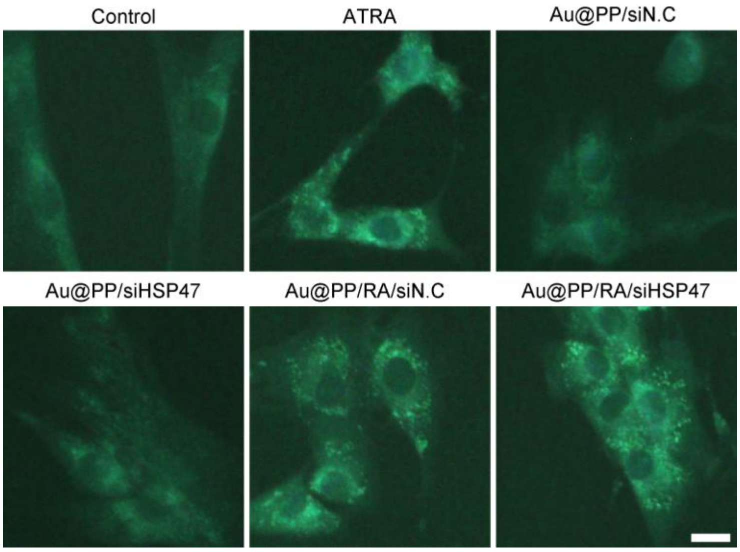

Supplementary Figure 11. Representative blue-green autofluorescence of retinol in PSCs after treatment with the indicated formulations for 48 h at pH 6.5. Scale bar, 20 μm. The fast-fading fluorescence excited at 328 nm exhibited the presence of vitamin A-containing lipid droplets in the cytoplasm.

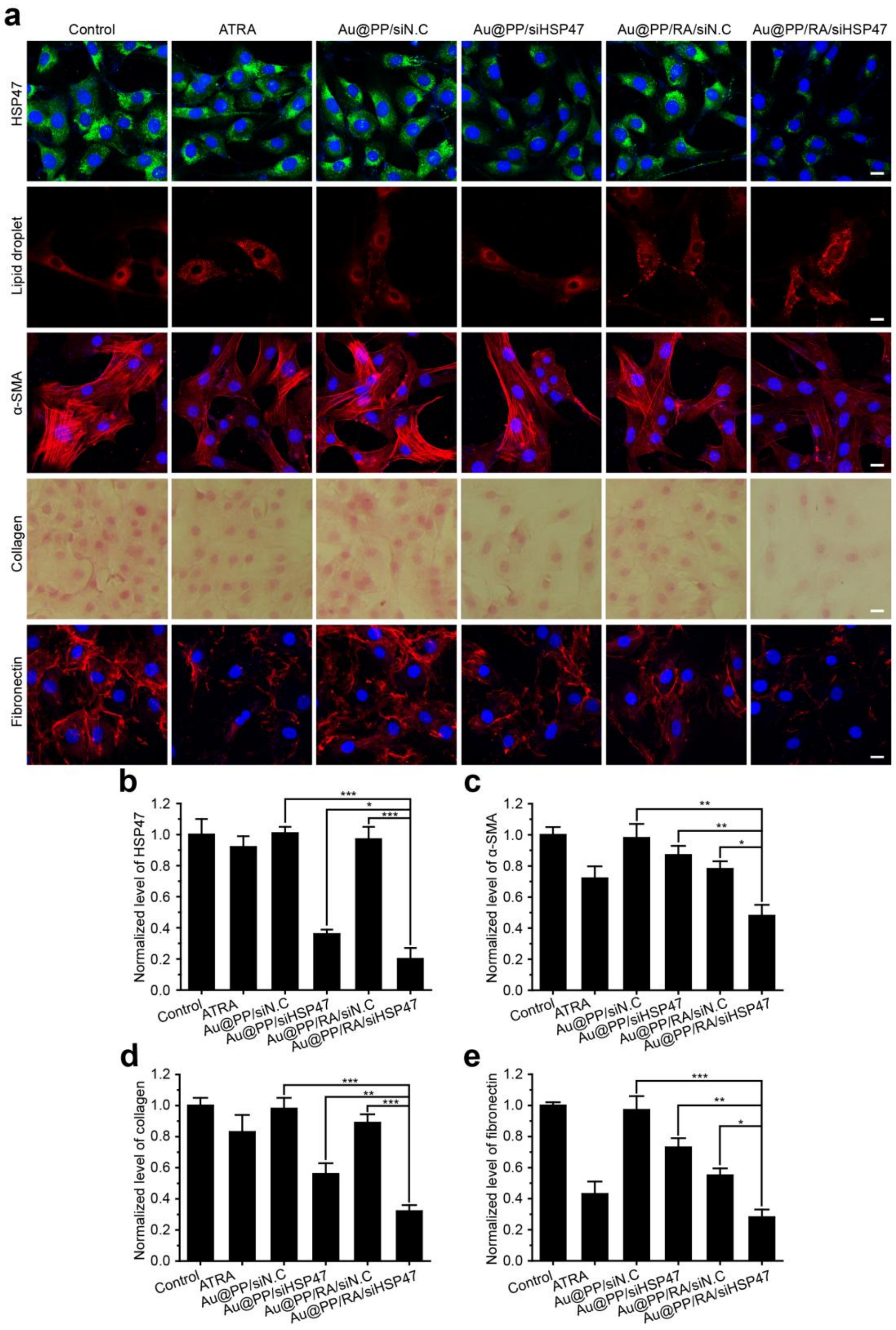

Supplementary Figure 12. Reversal of activated PSCs-2 and ECM reduction *in vitro*. (a) IF staining of HSP47, nile red staining of lipid droplets, IF staining of α-SMA, Sirius red staining of deposited collagen and IF staining of fibronectin in PSCs-2 after treatment with the indicated formulations for 48 h at pH 6.5. Scale bars, 20 μm. (b) Quantification of the normalized HSP47 protein expression (using Image J software). (c) Quantification of the normalized α-SMA protein

expression (using Image J software). (d) Normalized deposited collagen by measuring extracted Sirius red dye at 540 nm. (e) Quantification of the normalized fibronectin protein expression (using Image J software). The data are shown as the mean ± s.d. (n = 3). *$p < 0.05$, **$p < 0.01$, ***$p < 0.001$ (Student's *t*-test).

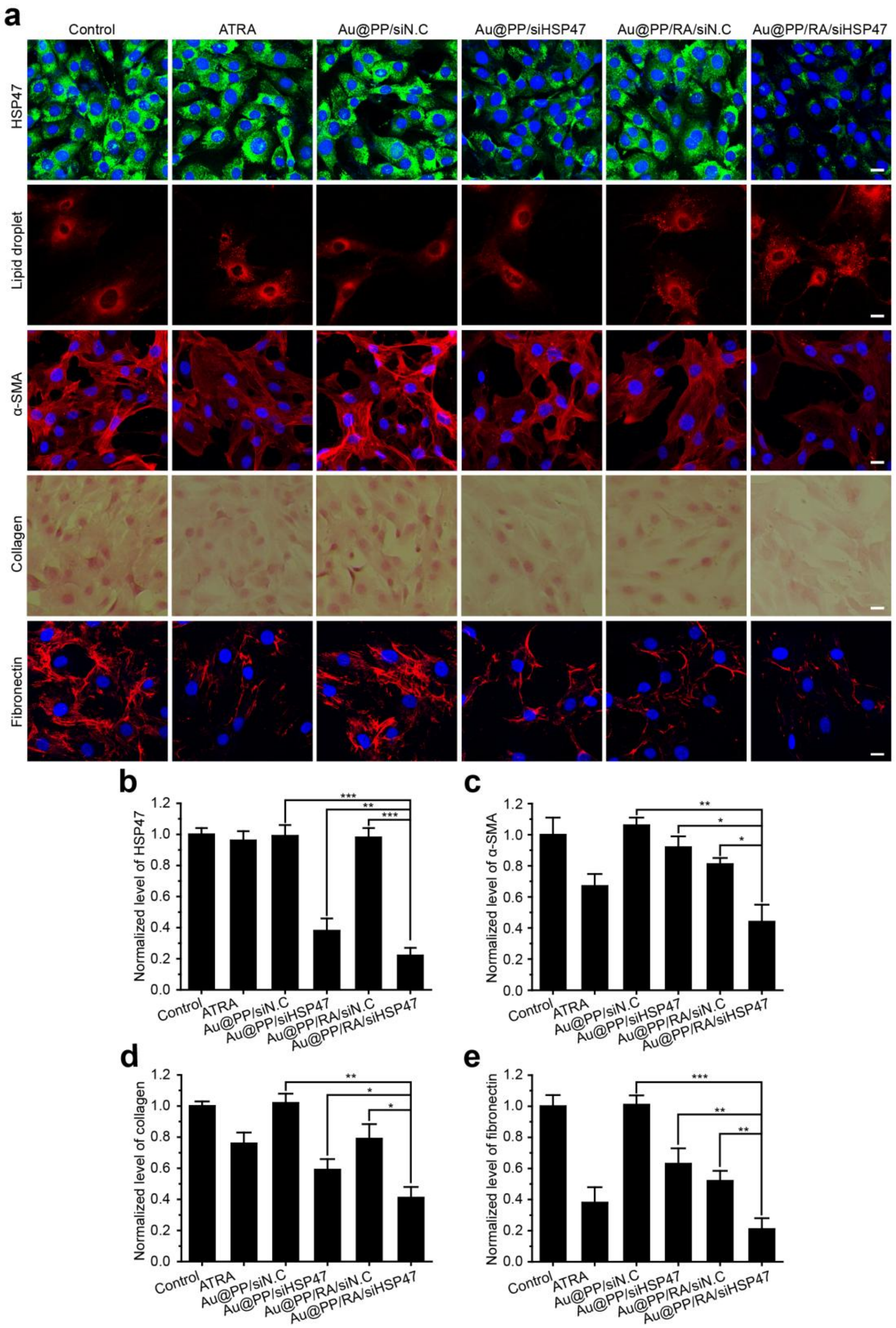

Supplementary Figure 13. Reversal of activated PSCs-3 and ECM reduction *in vitro*. (a) IF staining of HSP47, nile red staining of lipid droplets, IF staining of α-SMA, Sirius red staining of deposited collagen and IF staining of fibronectin in PSCs-3 after treatment with the indicated formulations for 48 h at pH 6.5. Scale bars, 20 μm. (b) Quantification of the normalized HSP47 protein expression (using Image J software). (c) Quantification of the normalized α-SMA protein

expression (using Image J software). (d) Normalized deposited collagen by measuring extracted Sirius red dye at 540 nm. (e) Quantification of the normalized fibronectin protein expression (using Image J software). The data are shown as the mean ± s.d. (n = 3). *$p < 0.05$, **$p < 0.01$, ***$p < 0.001$ (Student's *t*-test).

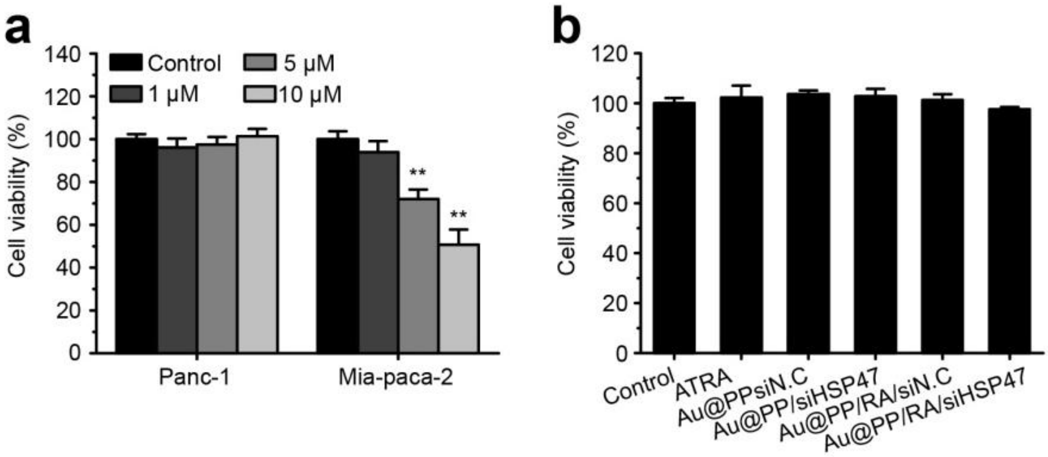

Supplementary Figure 14. Cell viability of Panc-1 and Mia-paca-2. (a) Panc-1 and Mia-paca-2 cells were incubated with different concentration of ATRA for 48 h and the cell viability was evaluated by CCK-8 assay. Mia-paca-2 cells were sensitive to ATRA, but Panc-1 cells were not. (b) Panc-1cells were treated with the indicated formulations at pH 6.5 for 48 h and the cell viability was evaluated by CCK-8 assay. The data are presented as the mean ± s.d. (n = 3). ***p* < 0.01 (Student's *t*-test).

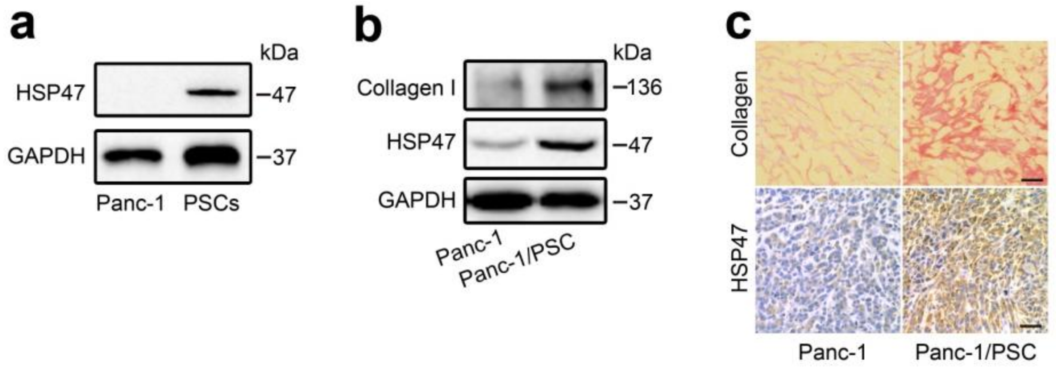

Supplementary Figure 15. Comparison of Panc-1 xenograft and Panc-1/PSC co-inoculated xenograft. (a) Western blot analysis of HSP47 expression in Panc-1 and PSCs. Expression of HSP47 was observed in PSCs but not in Panc-1 cells. (b) Western blot analysis of collagen I and HSP47 protein in Panc-1 and Panc-1/PSC tumour xenografts. (c) Representative Immunohistochemical staining images of HSP47 and Sirius red staining images of collagen in Panc-1 and Panc-1/PSC tumour xenografts. Panc-1/PSC tumour xenografts showed significantly greater desmoplasia compared to Panc-1 xenografts. Scale bars, 50 μm.

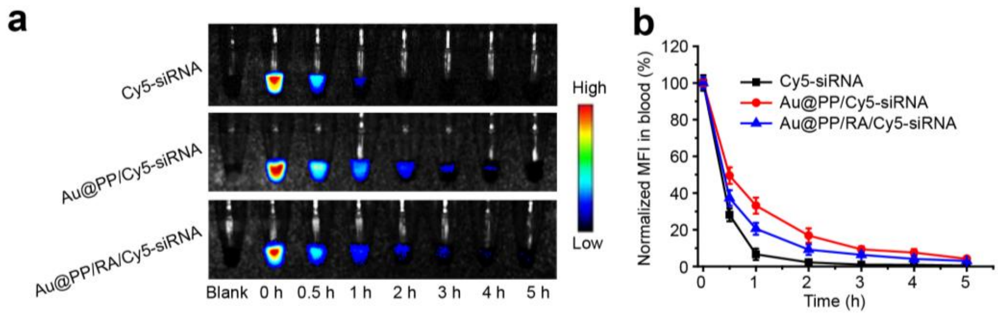

Supplementary Figure 16. The circulation time of Cy5-siRNA, Au@PP/Cy5-siRNA and Au@PP/RA/Cy5-siRNA in the blood. (a) Fluorescence images of blood drawn from mice at various time points after *i.v.* injection of Cy5-siRNA, Au@PP/Cy5-siRNA or Au@PP/RA/Cy5-siRNA. (b) Quantitative analysis of the normalized mean fluorescence intensity (MFI) in blood. The fluorescence intensity at 0 h in each group was normalized to 100%. The data are presented as the mean ± s.d. (n = 3).

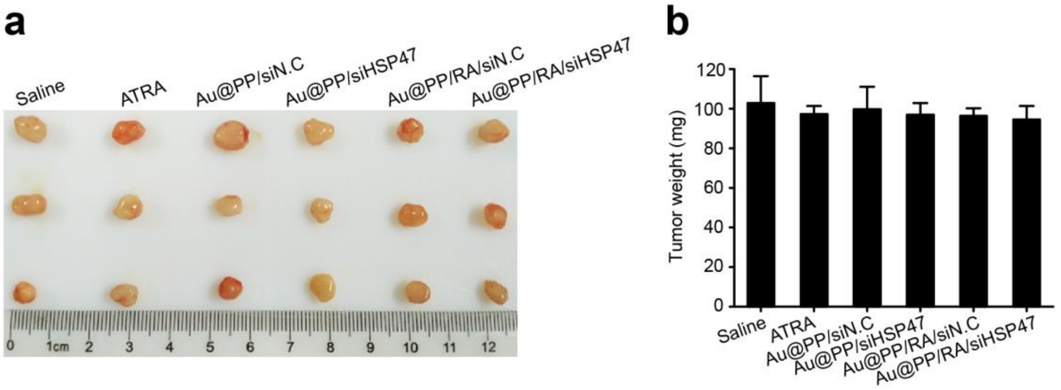

Supplementary Figure 17. Tumour weights of Panc-1/PSC xenografts after stroma modulation. (a) Images of excised tumours after different treatments. (b) The corresponding tumour weights of Panc-1/PSC xenografts in the indicated treatment groups. The data are presented as the mean $\pm$ s.d. (n = 3).

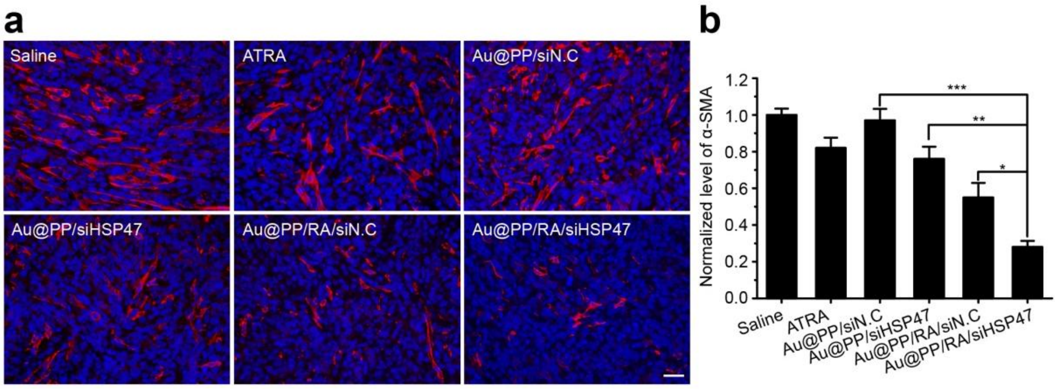

Supplementary Figure 18. Activated PSCs as evaluated by α-SMA staining of tumours. (a) Representative fluorescence images of α-SMA (red) in tumour slices after treatment with the indicated formulations. Nuclei were stained with Hoechst 33342 (blue). Scale bar, 50 μm. (b) Quantification of α-SMA using Image J software. The data are presented as the mean ± s.d. (n = 3). *p < 0.05, **p < 0.01, ***p < 0.001 (Student's t-test).

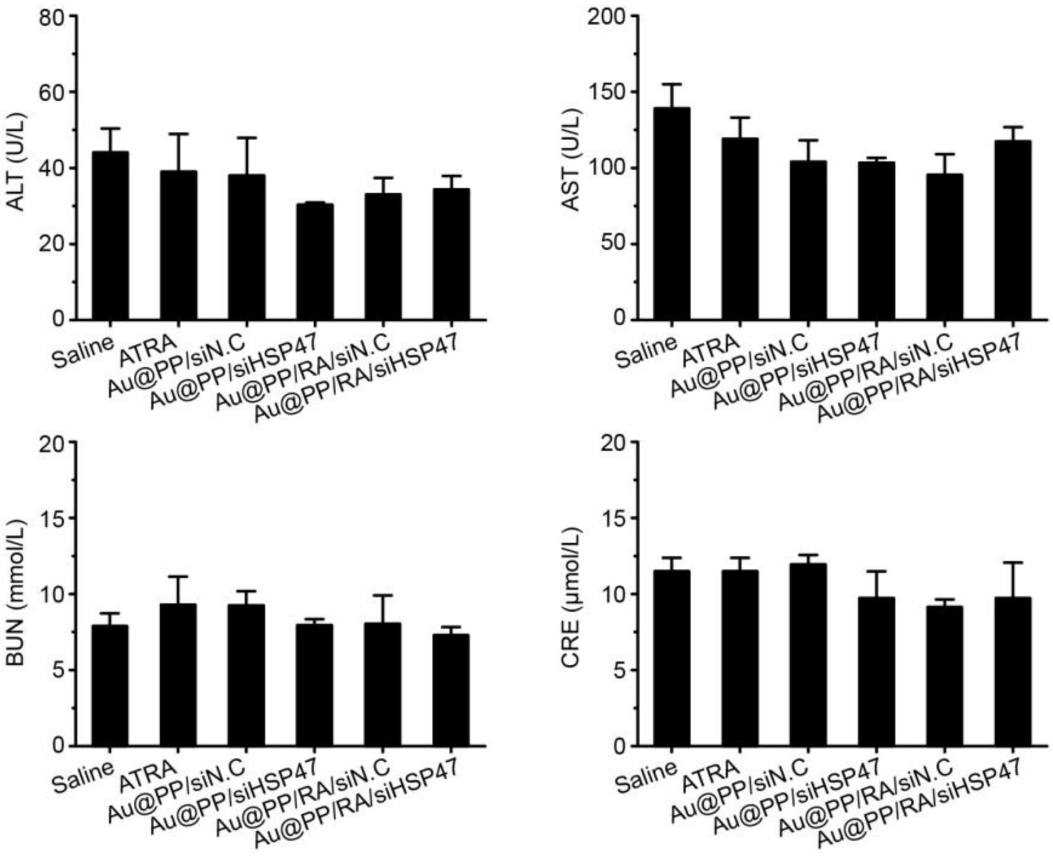

Supplementary Figure 19. Serum biochemistry of BALB/c nude mice after different treatments. ALT, alanine aminotransferase; AST, aspartate aminotransferase; BUN, blood urine nitrogen; CRE, creatinine. The data are presented as the mean ± s.d. (n = 3).

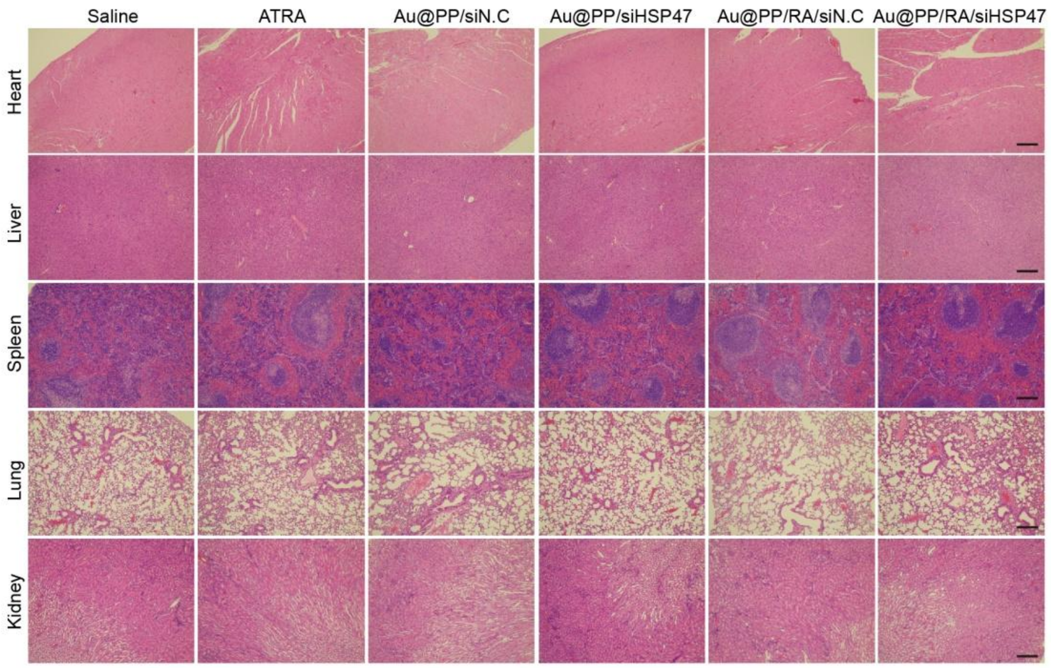

Supplementary Figure 20. H&E staining of major organs. No obvious morphological changes were observed after treatment with the indicated formulations. Scale bars, 100 μm.

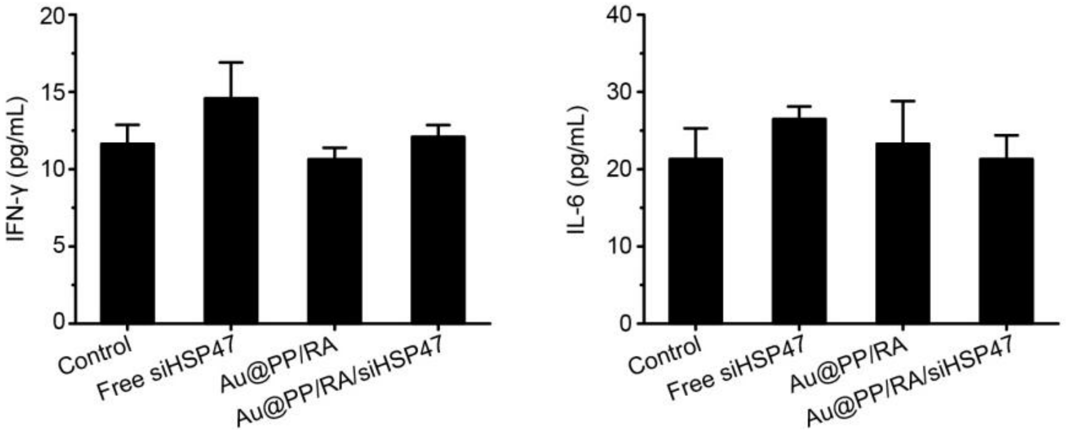

Supplementary Figure 21. Immunotoxicity analysis. IFN-γ and IL-6 concentrations were measured by ELISA in the serum of healthy BALB/c mice treated with the indicated formulations at 24 h post-injection. The data are presented as the mean ± s.d. (n = 3).

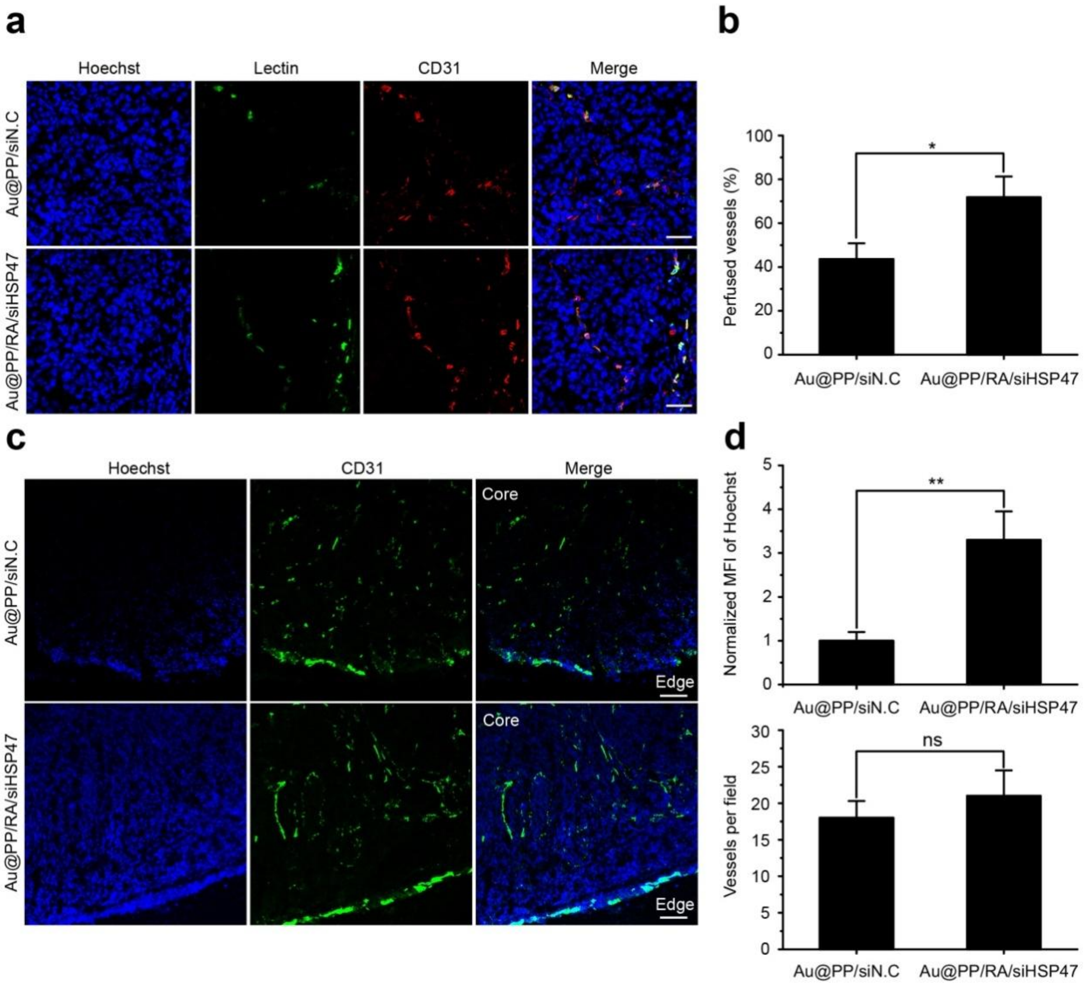

Supplementary Figure 22. Evaluation of tumour vessel perfusion and small molecule penetration. (a) Representative fluorescence images of lectin-FITC-perfused (green) and CD31-stained vessels (red) in tumour sections from Au@PP/siN.C- or Au@PP/RA/siHSP47-treated Panc-1/PSC xenografts. Nuclei were stained with Hoechst 33342 (blue). FITC-labeled lectin was injected into the vein tail and tumours were harvested at 10 min post-injection. Vessel perfusion was quantified in tumour cryosections after staining with the endothelial cell-specific marker CD31. Scale bars, 50 μm. (b) Quantification of perfusion ($CD31^{+}lectin^{+}$ area, percentage of total $CD31^{+}$ area). (c) Representative fluorescence images of penetrated Hoechst 33342 (blue) and CD31-stained vessels (green) in Panc-1/PSC tumours sections. Mice were injected with Hoechst 33342 *via* the tail vein 1 min before euthanasia. The tumour cryosections were made and stained with the endothelial cell-specific marker CD31. Scale bars, 100 μm. (d) Quantification of the normalized mean fluorescence intensity (MFI) of penetrated Hoechst 33342 (above, using Image J software) and quantification of the number of tumour vessels (below). The data are presented as the mean ± s.d. (n = 3). ns, no significant difference; $*p < 0.05$, $**p < 0.01$ (Student's *t*-test).

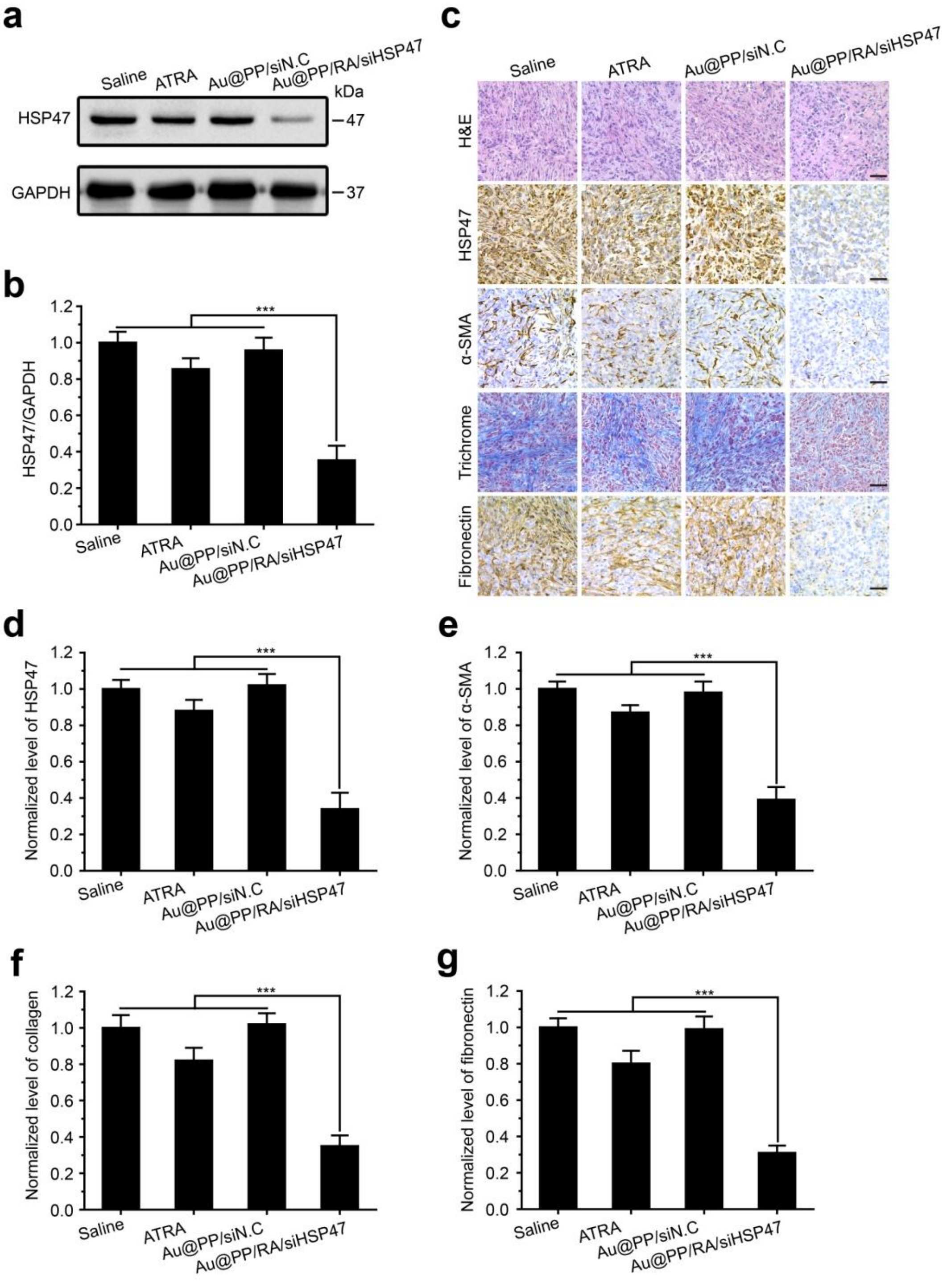

Supplementary Figure 23. Stroma modulation by Au@PP/RA/siHSP47 in Panc-1-luci/PSC orthotopic xenografts. (a) Western blot analysis of HSP47 protein in tumours. (b) Quantitative analysis of the normalized HSP47 protein expression in tumours (using Image J software). (c) Histological studies with H&E, trichrome staining of collagen and immunohistochemical staining of HSP47 and fibronectin in tumour sections. Scale bars, 50 μm. (d-g) Quantitative analysis of the normalized HSP47, α-SMA, collagen and fibronectin protein levels (using Image J software). The data are shown as the mean ± s.d. (n = 3). ***$p < 0.001$ (Student's *t*-test).

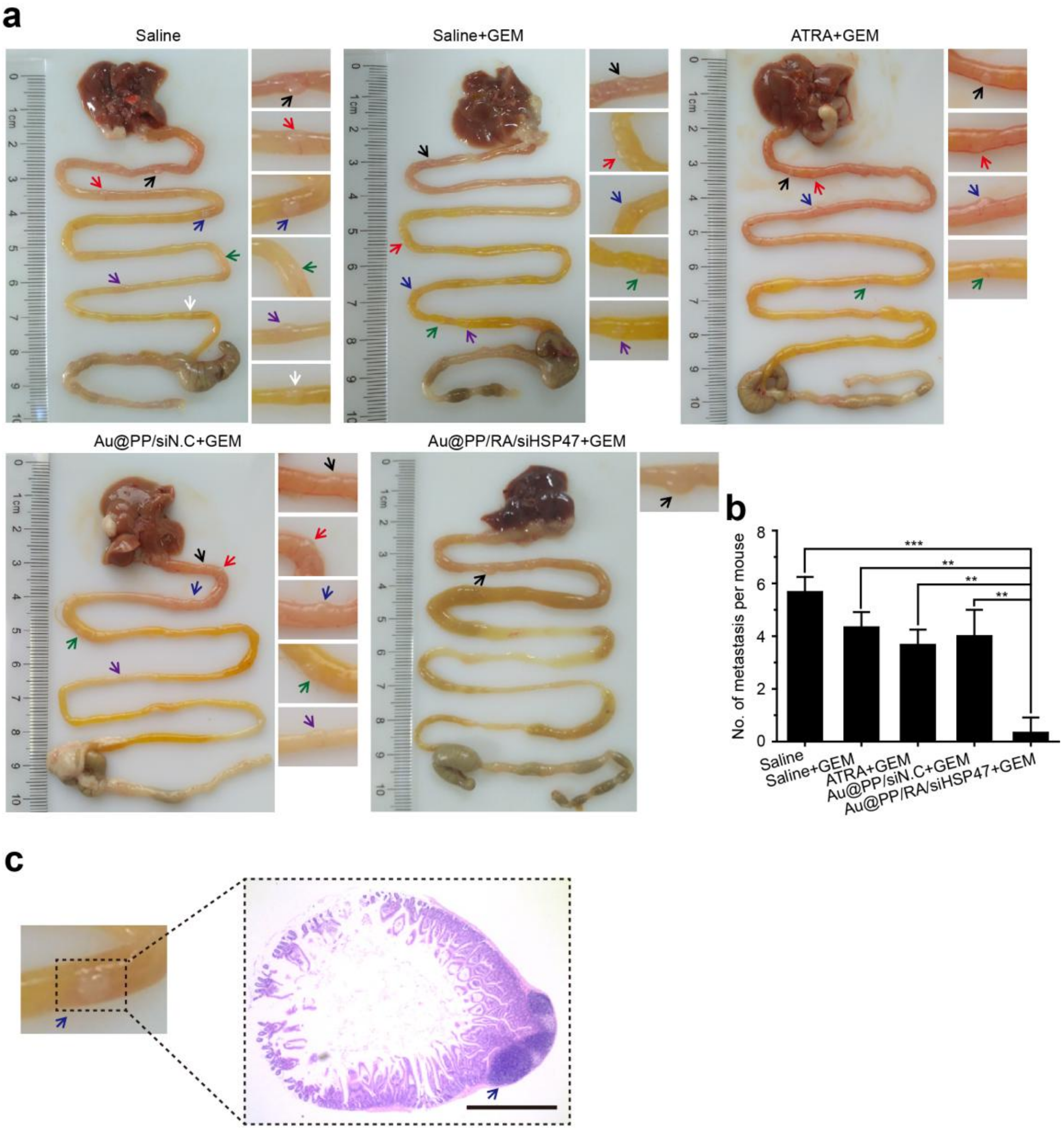

Supplementary Figure 24. Tumour metastasis foci of Panc-1-luci/PSC orthotopic pancreatic xenografts. (a) Representative images of tumour metastases (arrows) on the mesenteries. (b) Quantification of tumour metastases on mesenteries. The data are presented as the mean ± s.d. (n = 3). **p < 0.01, ***p < 0.001 (Student's t-test). (c) H&E-stained section of a representative nodule on mesentery of the saline group. Arrow indicates histopathologically confirmed tumour metastasis foci. Scale bar, 1 mm.

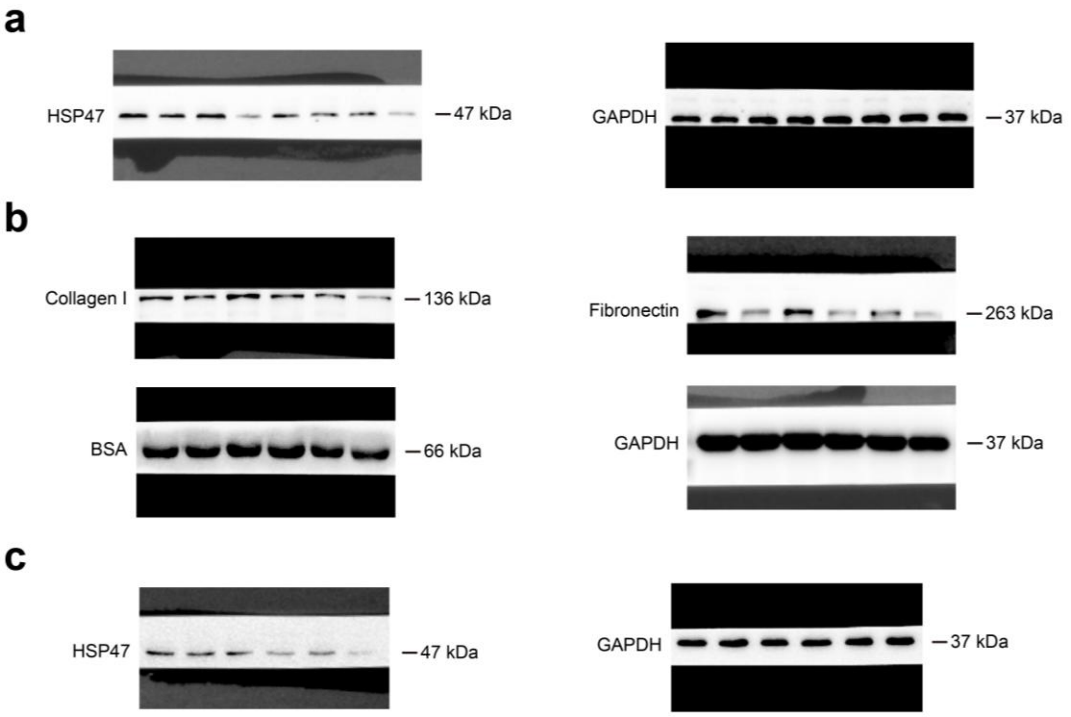

Supplementary Figure 25. Uncropped scans of blots. (a) Raw western blots form Figure 3d. (b) Raw western blots form Figure 4f. (c) Raw western blots form Figure 7d.
